# *Staphylococcus aureus* populations from the gut and the blood are not distinguished by virulence traits—a critical role of host barrier integrity

**DOI:** 10.1186/s40168-022-01419-4

**Published:** 2022-12-26

**Authors:** Elisa J. M. Raineri, Sandra Maaß, Min Wang, Siobhan Brushett, Laura M. Palma Medina, Neus Sampol Escandell, Dania Altulea, Erwin Raangs, Anne de Jong, Elias Vera Murguia, Edward J. Feil, Alex W. Friedrich, Girbe Buist, Dörte Becher, Silvia García-Cobos, Natacha Couto, Jan Maarten van Dijl

**Affiliations:** 1 Department of Medical Microbiology, University of Groningen, University Medical Center Groningen, Groningen, The Netherlands; 2 Department of Microbial Proteomics, Institute of Microbiology, University of Greifswald, Greifswald, Germany; 3 Present address: Department of Medicine Huddinge, Present Address: Center for Infectious Medicine, Karolinska Institute, Huddinge, Sweden; 4 Present address: Division of Nephrology, Department of Internal Medicine, University of Groningen, University Medical Center Groningen, Groningen, The Netherlands; 5 Department of Molecular Genetics, Groningen Biomolecular Sciences and Biotechnology Institute, University of Groningen, Groningen, The Netherlands; 6 Department of Biology and Biochemistry, The Milner Centre for Evolution, University of Bath, Bath, UK; 7 Present address: Reference and Research Laboratory On Antimicrobial Resistance and Healthcare Associated Infections, Centro Nacional de Microbiología, Instituto de Salud Carlos III (ISCIII), Madrid, Spain

**Keywords:** S. aureus, Gut, Enteric carriage, Bacteremia, Barrier, Virulence

## Abstract

**Background:**

The opportunistic pathogen *Staphylococcus aureus* is an asymptomatically carried member of the microbiome of about one third of the human population at any given point in time. Body sites known to harbor *S. aureus* are the skin, nasopharynx, and gut. In particular, the mechanisms allowing *S. aureus* to pass the gut epithelial barrier and to invade the bloodstream were so far poorly understood. Therefore, the objective of our present study was to investigate the extent to which genetic differences between enteric *S. aureus* isolates and isolates that caused serious bloodstream infections contribute to the likelihood of invasive disease.

**Results:**

Here, we present genome-wide association studies (GWAS) that compare the genome sequences of 69 *S. aureus* isolates from enteric carriage by healthy volunteers and 95 isolates from bloodstream infections. We complement our GWAS results with a detailed characterization of the cellular and extracellular proteomes of the representative gut and bloodstream isolates, and by assaying the virulence of these isolates with infection models based on human gut epithelial cells, human blood cells, and a small animal infection model. Intriguingly, our results show that enteric and bloodstream isolates with the same sequence type (ST1 or ST5) are very similar to each other at the genomic and proteomic levels. Nonetheless, bloodstream isolates are not necessarily associated with an invasive profile. Furthermore, we show that the main decisive factor preventing infection of gut epithelial cells in vitro is the presence of a tight barrier.

**Conclusions:**

Our data show that virulence is a highly variable trait, even within a single clone. Importantly, however, there is no evidence that blood stream isolates possess a higher virulence potential than those from the enteric carriage. In fact, some gut isolates from healthy carriers were more virulent than bloodstream isolates. Based on our present observations, we propose that the integrity of the gut epithelial layer, rather than the pathogenic potential of the investigated enteric *S. aureus* isolates, determines whether staphylococci from the gut microbiome will become invasive pathogens.

Video Abstract

**Supplementary Information:**

The online version contains supplementary material available at 10.1186/s40168-022-01419-4.

## Background

*Staphylococcus aureus* is an omnipresent member of the human microbiome that is infamous for causing a broad range of potentially life-threatening infections and for its high capacity to acquire multi-drug resistance [40, 64, 81]. This is critically underscored by the methicillin-resistant *S. aureus* (MRSA) lineages that have emerged in hospitals and the community [39, 50, 51, 82]. Moreover, *S. aureus* is able to persist in the human host causing chronic recurrent infections [31, 64, 80]. Nonetheless, the microbiome of about one third of the human population include *S. aureus* asymptomatically over prolonged periods of time [20, 21, 49, 65, 70, 85].

In view of the difficulties encountered in the treatment of staphylococcal infections, it is important to understand what factors determine the transition from asymptomatic colonizer to the invasive pathogen [6, 10, 52, 67, 70]. It is unknown to what extent this transition is explained by genetically derived variation in virulence potential between different *S. aureus* strains. Different epidemiological features, adherence to the host’s skin and mucosa, as well as cross-talk with the host’s immune defenses are major elements in the pathogenicity of *S. aureus* [54, 70]. Moreover, the ability of *S. aureus* to hide in protective extra- or intracellular niches is a key determinant for recurrent infections. Remarkably, *S. aureus* is even able to survive within phagocytic immune cells and it can use these as Trojan horses to reach, colonize, and invade different sites within the human body [31, 35, 37, 67, 76, 80, 90]. To do so, *S. aureus* has evolved a plethora of adaptive strategies, which allow this pathogen to overcome the physical, immunological, and chemical barriers imposed by the host and to become an integral member of the microbiota of many individuals [36, 77]. Conversely, the responses to invading staphylococci differ between sites within the host, depending on the integrity of primary epithelial or endothelial barriers and homeostasis of the microbiota [47, 62, 68, 76].

The most well-characterized *S. aureus* reservoirs in the human body are the nasal and oral cavities and the skin [34, 36, 54, 58, 85]. The eradication of *S. aureus* from these sites has become an effective element in the prevention of post-surgical wound, implant, and bloodstream infections [3, 18]. However, in recent years, evidence has accumulated for the existence of gastrointestinal *S. aureus* reservoirs [2, 8, 20, 21, 67]. In fact, the frequency of *S. aureus* intestinal carriage in healthy individuals and patients may amount ∼25%, with higher prevalence during the early stages of life or in patients with skin and soft tissue infections [21]. Accordingly, intestinal *S. aureus* carriage was implicated as a potential player in the community- or hospital-acquired infections [8, 20, 21, 35, 71, 90].

The mechanisms that allow *S. aureus* to breach the gut barrier and to disseminate into the underlying tissues and the bloodstream remain poorly understood [67]. The main objective of the present study was to investigate the extent to which genetic differences between enteric *S. aureus* isolates and isolates that have caused serious bloodstream infections contribute to the likelihood of invasive disease. To this end, 69 gut isolates (GI) from a cohort of healthy individuals and 95 blood-culture isolates (BI) from patients with bacteremia were characterized by whole-genome sequencing, and these data were analyzed using genome-wide association studies (GWAS). Subsequently, the cellular and extracellular proteomes of representative GI and BI isolates were investigated by mass spectrometry (MS) to distinguish potentially distinctive features in metabolic adaptation or virulence. Lastly, the virulence potential of the selected GI and BI isolates was evaluated using infection models based on human gut epithelial cells, blood cells from healthy donors, and a small animal infection model. Intriguingly, our results show that GI and BI isolates with the same sequence type (ST1 or ST5) are very similar to each other at the genomic and proteomic levels. Nonetheless, this is not necessarily associated with an invasive profile. Furthermore, we show that the main decisive factor preventing infection of gut epithelial cells in vitro is the presence of a tight barrier. Based on our present observations, we propose that the integrity of the gut epithelial layer, rather than the pathogenic potential of the investigated enteric *S. aureus* isolates, determines whether transiently carried or colonizing staphylococci will become invasive pathogens.

### Methods

#### Sample collection and whole-genome sequencing

Sixty-nine methicillin-susceptible *S. aureus* (MSSA) GI isolates were retrieved in a single-screening event from stool samples of 69 healthy volunteers from the Northern Netherlands participating in the LifeLines cohort study [75] (https://www.lifelines.nl/). Ninety-five MSSA BI isolates were retrieved in the years 2014 and 2016–2018 from blood cultures of 95 patients with puncture- or line infection-related bacteremia at the University Medical Center Groningen (UMCG). Metadata concerning the respective patient characteristics was not retained based on medical-ethical considerations.

DNA extraction was performed with the DNeasy UltraClean microbial kit (Qiagen, Hilden, Germany). DNA concentrations were measured with a Qubit 2.0 fluorometer (Life Technologies, Thermo Fisher Scientific, Waltham, MA). A pooled DNA library was prepared with the Nextera DNA Flex Library Prep kit, using 2 μL with 100–500 ng of DNA for each isolate (Illumina, California, USA), in accordance with the manufacturer’s protocol. Sequencing was performed with the NextSeq platform (Illumina), generating 2 × 150-bp reads. Six selected strains were also sequenced using the MinION platform (Oxford Nanopore Technologies [ONT], Oxford, UK) and the Rapid Barcoding Sequencing kit (SQK-RBK004) and a FLO-MIN106 R9 flowcell (ONT). Base-calling was performed with Guppy v3.2.10 (ONT).

#### Bioinformatics for genome analysis

Reads were trimmed and assembled through Shovill v1.1.0 (with the trim option, https://github.com/tseemann/shovill) using Trimmomatic v0.39 and SPAdes v3.14.1 for trimming and assembly, respectively. The assemblies were annotated with Prokka v1.14.6. Variants were called through Snippy v4.6.0 (https://github.com/tseemann/snippy) using as a reference the annotated *S. aureus* SBB155 genome (GenBank accession number LN854556). The default parameters were used (snippy_multi) that assume a minimum number of reads covering a site of 10 reads, a minimum mapping quality of 60, and a minimum base quality of 13 (error probability ~ 5%). We constructed an approximate maximum likelihood phylogeny using FastTree v2.1.10 [66] assuming a general time reversible model after running Gubbins v2.4.1 [14] to remove recombination and SNP-sites v2.5.1 [61]. The tree was visualized with Microreact v178.0.0 [4]. Annotated assemblies were run through Roary v3.13.0 [60] with the option to not split paralogs to define the core and pangenomes. Piggy v1.5 [79] was used to analyze the intergenic regions (IGRs) of the genomes. To identify clusters based on the pangenome, we used Panini v1.7.1 [1] and Poppunk v2.4.0 using the DBSCAN model [42]. Microreact v178.0.0 [4] was used for visualization. ONT reads were demultiplexed and trimmed with Porechop v0.2.4 (https://github.com/rrwick/Porechop). The Illumina and ONT reads were assembled using Unicycler v0.4.8-beta [86]. RAST v2.0 [5] was used for annotation of the hybrid assembled contigs.

We used three different approaches for the GWAS studies. In the first one, the results from Roary and Piggy were run through Scoary v1.6.16 [11] to identify genes or IGRs associated with the two different traits (carriage versus infection). Scoary is a tool to find associations between the presence or absence of pan-genome genes/IGRs and a certain phenotype or trait [11]. *P* values were corrected for multiple comparisons using the Bonferroni correction and were considered significant if *P* < 0.05. These results were also validated using the linear fixed effects model implemented in Pyseer v1.3.9 [41], while controlling for population structure using a mash distance matrix. In the second approach, the SNPs (core.vcf) identified through Snippy in the approach mentioned above were run through Pyseer v1.3.9 [41] to find SNPs associated with traits. The association between each SNP with the phenotype was tested controlling for population structure (based on a kinship matrix K produced by using the similarity_pyseer –vcf script in Pyseer and the core.vcf generated by Snippy) using the linear-mixed model (LMM) implemented in Pyseer [41]. We chose the LMM model because, in exchange for reduced power when compared to the fixed model or the elastic net model, the LMM offers the best control of false positives [43]. For genetic variants covering the entire pangenome (third approach), we used unitigs instead of k-mers, since the former has been shown to reduce redundancy [32]. To call and count unitigs, we used unitig-counter v1.1.0 ( [32],https://github.com/johnlees/unitig-counter). Afterwards, the association between each unitig and the phenotype (carriage versus infection) was performed in the same way as for SNPs. *P* values for both approaches were considered significant if *P* was smaller than α/np, where *α* = 0.05 and np is the number of (SNPs or unitigs) patterns observed. The results were visualized in Rstudio v1.4.1106 using the ggplot2, dplyr, tidyverse, ggrepel, and ggtext packages.

#### Bacterial cultivation

Six representative *S. aureus* BI and GI isolates with ST1 (denoted BI-ST1-8, GI-ST1-7, and GI-ST1-9) or ST5 (denoted BI-ST5-1, GI-ST5-4, GI-ST5-6) were characterized by MS-based analyses of the cellular and extracellular proteomes and infection experiments. To monitor infection by flow cytometry or fluorescence microscopy, these *S. aureus* isolates were transformed with plasmid pJL-sar-GFP_redopt-cm in order to express the green fluorescent protein (GFP; [68]. The resulting strains were grown overnight at 37 °C in tryptic soy broth (TSB; OXOID, Basingstoke, UK) under constant shaking (250 rpm). Chloramphenicol (10 µg·mL^−1^) was added to prevent the possible loss of pJL-sar-GFP_redopt-cm. The overnight cultures were used to inoculate pre-cultures and, subsequently, main cultures in Roswell Park Memorial Institute 1640 medium (RPMI) (Gibco, New York), as detailed in the following sections. In the present studies, the RPMI medium was invariably supplemented with 2 mM L-glutamine (Thermo Fisher Scientific, Waltham, USA).

#### Sample preparation for MS/MS analysis of proteome samples

*S. aureus* strains were grown overnight at 37 °C in TSB under vigorous shaking (250 rpm). The cultures were then diluted into 25 mL pre-warmed RPMI medium to an optical density at 600 nm (OD_**600**_) of 0.1 and cultivation was continued at 37 °C under vigorous shaking (115 rpm) in a water bath. At an OD_**600**_ of ~ 0.5, cultures with exponentially growing bacteria were diluted into 100 mL of fresh pre-warmed RPMI medium to a final OD_**600**_ of 0.1. The bacterial cultivation was continued until an OD_**600**_ of ~ 1.2 was reached, which corresponds to the stationary growth phase. The bacterial cells were then separated from the growth medium by centrifugation. For analysis of the extracellular proteome, proteins in the growth medium were enriched by primed affinity bead purification with StrataClean beads (Agilent, Santa Clara, USA) and subsequently eluted from the beads by polyacrylamide gel electrophoresis (PAGE) using 4–20% Criterion TGX gels (Bio-Rad, Feldkirchen, Germany) [9]. For analysis of the cellular proteome, the collected cells were washed once with TE buffer (10 mM Tris, 5 mM EDTA, pH7.5). Subsequently, the cells were resuspended in TE buffer and disrupted with glass beads using a Precellys24 bead beater (Bertin Technologies, Montigny-le-Bretonneux, France). Lastly, cellular debris was removed by centrifugation and the cellular proteins in the supernatant fraction were collected and purified by PAGE using 4–20% criterion TGX gels.

Following PAGE with a 1-cm separating distance for extracellular proteins and a complete separation for cellular samples, proteins were in-gel digested with trypsin (Promega, Madison, USA) and prepared for MS/MS ac [9]. Briefly, gel lanes were cut resulting in one or ten gel pieces per sample for extracellular or cellular proteins, respectively. Gel pieces were sliced into smaller blocks and transferred into low-binding tubes where samples were destained and dried in a vacuum centrifuge before being covered with trypsin solution. Digestion was carried out at 37 °C overnight before peptides were eluted in water by ultrasonication. The peptide-containing supernatant was transferred into a fresh tube and desiccated in a vacuum centrifuge. The peptides were then resolubilized in 0.1% (v/v) acetic acid for MS analyses.

#### MS/MS analysis of proteome samples

Tryptic peptides were separated with an EASY-nLC II liquid chromatography (LC) system (Thermo Fisher Scientific, Waltham, Massashussets, USA) using self-packed analytical columns (OD 360 μm, ID 100 μm, length 20 cm) filled with 3-µm diameter C18 particles (Dr. Maisch, Ammerbuch-Entringen, Germany). The peptides were eluted using a binary nonlinear gradient of 5–99% acetonitrile in 0.1% acetic acid over 151 min (secreted proteins) or 76 min (cellular proteins) with a flow rate of 300 nL/min. Subsequently, peptides were subjected to electrospray ionization-based MS/MS with an LTQ Orbitrap XL (Thermo Fisher Scientific) to identify secreted proteins or with an LTQ Orbitrap (Thermo Fisher Scientific) to identify cellular proteins. After a survey scan at a resolution of 30,000 in the Orbitrap using lockmass correction, the five most abundant precursor ions were selected for fragmentation. Singly charged ions, as well as ions without detected charge states, were not selected for MS/MS analysis. Collision-induced dissociation fragmentation was performed for 30 ms with a normalized collision energy of 35, and the fragment ions were recorded in the linear ion trap.

#### MS data analysis

The raw MS data for extracellular and cellular proteins were processed separately using MaxQuant v1.6.17.0 [83, 84]. Database searches were performed against a non-redundant database of all unique proteins of *S. aureus* isolates containing 10,771 entries with common contaminants and reserve entries added by MaxQuant. The non-redundant database was created by combining all proteins of all isolates and subsequent removal of the redundant proteins. A BLAST search against the UniProt database [78] was done to add UniProt IDs to proteins if the *E* value was < 10E − 09. The maximum number of allowed missed cleavages was 2, and precursor mass tolerance was set to 4.5 ppm. Methionine oxidation and acetylation (protein N-termini) were set as variable modifications for both datasets. A match between runs was applied, and protein abundances were calculated based on label-free quantification intensities (LFQ) using the MaxLFQ algorithm [13]. Identified protein groups were analyzed with Perseus v1.6.14.0 [83, 84]. A protein was only considered for further analysis if a minimum of two unique peptides per protein was identified and if the protein was quantified in at least two out of three biological replicates.

#### Prediction of protein localization, biological processes, and molecular functions

Prediction of the subcellular localization of proteins that were identified by LC–MS/MS was performed using the GP4 Gram Positive Protein Prediction Pipeline [23]. Gene annotations and functional categories were assigned using TIGRfam (v 15.0) and the AureoWiki database [16]. Voronoi treemaps to link quantitative proteomics data and functional categories were created using the Paver software v2.1 (DECODON, Greifswald, Germany) [45]. Proteins were assigned to regulons using the RegPrecise 3.0 database, the SEED database, and the AureoWiki database [16, 55, 56, 59]. Additionally, cellular proteins were assigned to metabolic pathways using the SEED database and the AureoWiki database [16, 51, 59, 63].

#### Statistical analyses

Individual proteins identified by MS were considered as differentially expressed if their LFQ intensities showed a fold change of |0.8|, and the *P* value was < 0.01 as assessed by ANOVA. Significant differences in the log2-transformed LFQ intensities per sequence type (ST5 versus ST1) or isolation site (GI versus BI) were assessed by Student’s *t* tests and subsequent Holm-Sidak, Bonferroni-Dunn, and Sidak-Bonferroni corrections to adjust the *P* values using GraphPad Prism v8. (GraphPad Software, San Diego, CA, USA). In this case, a *P* value < 0.05 was considered significant.

To investigate the relationships between the *S. aureus* isolates in terms of their extracellular and cellular proteome profiles, principal component analysis (PCA) and graphical representation in heatmaps were performed using ClustVis [53]. To visualize differences in identified virulence factors, extracellular cytoplasmic proteins (ECPs), and metabolic pathways, heatmaps were created using GraphPad Prism v8 (GraphPad Software). Venn diagrams were drawn using the web-based tool “InteractiVenn” [27].

#### Caco2 infection experiments and flow cytometry

The human colon adenocarcinoma cell line Caco2 (ATCC) was cultured in Dulbeco’s modified Eagle’s medium (DMEM; Thermo Scientific, Waltham, MA, USA), supplemented with 10% fetal bovine serum (FBS; Sigma-Aldrich, St. Louis, MO, USA) and 1% nonessential amino acids (NEAA; Life Technologies, Carlsbad, CA, USA), using T75 flasks (TPP, Switzerland). The medium was changed every 48 h. In all experiments, Caco2 cells between passages 10 and 25 were used.

For infection experiments, Caco2 cells were seeded in 24-well plates (Greiner, Germany) at a density of 200,000 cells per well and cultured for 84 h prior to infection. The numbers of cells were counted prior to infection with *S. aureus*. A multiplicity of infection (MOI) of 30 was used for all internalization experiments. The bacterial master mix for infection was prepared from stationary growing *S. aureus* cells in RPMI (OD_600_ of 1.2) and resuspended in Caco2 culture media. The bacterial master mix was added to the Caco2 cells, and subsequently, the infected cells were incubated for 3 h (37 °C, 5% CO_2_). Two sets of samples were collected. To analyze all cells that contained either attached or internalized bacteria or that were not infected, a first set of samples was collected immediately after infection and the cells were then fixed with 4% formaldehyde for 15 min at room temperature and resuspended in phosphate-buffered saline (PBS). To analyze only the cells that contained internalized bacteria or that were not infected, for the second set of samples, the medium was removed and replaced with Caco2 media containing 25 µg·mL^−1^ of lysostaphin (AMBI Products, New York). Incubation was then continued for 30 min to eliminate all non-internalized bacteria bound to the Caco2 surface. The cells were then fixed with 4% formaldehyde for 15 min at room temperature and resuspended in PBS.

The numbers of Caco2 cells were counted by flow cytometry. To this end, the cells were treated with trypsin–EDTA (Thermo Fisher Scientific, the Netherlands) during a 10-min incubation at 37 °C, 5% CO_2_. Counting of the infected Caco2 cells was then performed with a Cytoflex S flow cytometer (Beckman Coulter, Woerden, the Netherlands) by excitation of GFP with a 488-nm laser and detection at 525/40 nm. Analysis of the flow cytometry data was performed using Kaluza Analysis Software (Beckman Coulter, Woerden, The Netherlands). A gating strategy was applied to distinguish healthy cells from cellular debris. Distributions and median-mean values of 20,000 cells were thus obtained. The relative numbers of infected Caco2 cells were expressed as the percentage of GFP-positive cells compared to the uninfected control cells.

To investigate the influence of tight junctions between the Caco2 cells on bacterial internalization, a third set of samples was included in the analyses where the tight junctions were disrupted by treatment of the cells with EGTA (Sigma-Aldrich, St. Louis, Missouri, USA). In this case, the Caco2 cells were seeded under the same conditions as detailed above, but incubated with 5 mM EGTA for 45 min in a calcium-free DMEM medium prior to infection. Subsequently, the cells were infected under the same conditions as above, but with a master mix of *S. aureus* in calcium-free DMEM medium supplemented with 10% fetal bovine serum and 1% non-essential amino acids. For flow cytometry, the medium was removed and replaced with DMEM medium containing 25 µg·mL^−1^ of lysostaphin. Incubation was continued for 30 min to eliminate non-internalized bacteria bound to the Caco2 surface. The cells were then fixed with 4% formaldehyde for 15 min at room temperature and resuspended in PBS.

#### Fluorescence microscopy of infected Caco2 cells

Immunofluorescence microscopy was performed using a Leica TCS SP8 Confocal laser scanning microscope (Leica Microsystems, Wetzlar, Germany). Caco2 cells were seeded under the same conditions as described above, but in this case on top of round coverslips with a diameter of 13 mm #1.5 (Thermo Fisher Scientific, Waltham, USA). Coverslips with infected or uninfected cells were collected at different time points and fixed with 4% formaldehyde for 15 min at room temperature. Subsequently, the cells were permeabilized, and blocked to avoid non-specific antibody binding, by incubation for 20 min at room temperature with 0.5% Tween-20 in PBS, followed by overnight incubation at 4 °C with 2% BSA and 5% neutral goat serum in PBS. Additional blocking was performed by incubation with 12 µg/ml of the human monoclonal antibody 6D4 [29], diluted in the same blocking solution, for 2 h at room temperature in a humidified chamber. Tight junction proteins were immunostained to visualize their expression and distribution inside the cells by confocal microscopy. The fixation, permeabilization, and blocking procedures were carried out as described above. Subsequently, cells were incubated for 1 h at room temperature with a polyclonal rabbit primary antibody against the tight junction protein zonula occludens-1 (ZO-1; Life Technologies, NY, USA) at a dilution of 1:100. The bound antibody was visualized by incubation for 1 h with a secondary donkey anti-rabbit antibody conjugated with Alexa Fluor 647 (Life technologies, NY, USA) at a 1:500 dilution. Lastly, DNA was stained with 4′,6-diamidino2-phenylindole (DAPI; Roche, Switzerland). The slides were mounted with Mowiol 4–88 (Merk Millipore,USA) and stored at − 20 °C until microscopic visualization. Image processing was performed using FIJI (https://fiji.sc/).

#### Blood cell infection experiments and flow cytometry

The blood was collected from healthy volunteers in hirudin-coated tubes (S-Monovette, Sarstedt), and the red blood cells were lysed using ammonium chloride lysis buffer (NH_4_Cl 8.3 g/L, KHCO3 1.0 g/L, disodium EDTA dihydrate 37 mg/L, UMCG hospital pharmacy). For every 100 µL of the blood, 1 mL of the lysis buffer was added. The mixture was incubated for 5 min by gentle shaking on ice and followed by centrifugation (5 min, 1000 rcf, 4 °C). These two steps were repeated once to obtain a pellet of purified immune cells, which was resuspended in the RPMI medium. Subsequently, the number of blood cells was counted (by flow cytometry and by cell counter) and a MOI of 15 was used for all infection experiments. A bacterial master mix for infection was prepared from the main culture of *S. aureus* cells grown to the stationary phase (OD_600_ of 1.2) in RPMI. The bacterial master mix was added to the blood cells, and the infected cells were incubated for 30 min (37 °C, 5% CO_2_). Afterwards, 25 µg·mL^−1^ of lysostaphin was added to the samples to eliminate non-internalized bacteria bound to the blood cells’ surface and incubation was continued for 30 min (37 °C, 5% CO_2_). The cells were then fixed with 4% formaldehyde for 15 min at room temperature and resuspended in PBS (Gibco Waltham, Massachusetts, USA). The number of blood cells was measured by flow cytometry. For this purpose, samples were collected in triplicate and counting of infected blood cells was performed with a BD LSR-II flow cytometer (BD biosciences, USA) by excitation of GFP with a 488-nm laser and detection at 530/30 nm. For the analysis, 30,000 events per sample were counted and the data were analyzed with Kaluza Analysis Software (Beckman Coulter, Woerden, the Netherlands). A gating strategy was applied to distinguish monocytes and granulocytes from lymphocytes and debris. The relative number of infected blood cells was expressed as the percentage of living cells post-infection (p.i.) compared to the control sample with uninfected cells. Additionally, the relative number of infected granulocytes (GFP-positive or -negative) was expressed as the percentage of living cells p.i. compared to the control sample with uninfected cells.

#### Assessment of S. aureus virulence in a Galleria mellonella larval infection model

*G. mellonella* larvae in their final instar stage (Frits Kuiper, Groningen, The Netherlands) were maintained on wood chips in the dark and used within 7 days of receipt. Larvae of ~ 250 mg in weight and 2 cm in length were employed in all assays. Fifteen randomly selected larvae were used for assessing the virulence of each investigated *S. aureus* isolate, with each infection experiment being repeated at least 3 times. Before inoculation of the larvae, *S. aureus* cells were harvested in the exponential growth phase (OD_600_ of 0.4) from a culture in RPMI. An insulin pen (HumaPen LUXURA® HD, Indianapolis, USA) was used to inject 10-μL aliquots of a diluted bacterial suspension (1 × 10^8^ CFU/mL) in PBS into the hemocoel via the last pro-leg. Two control groups were used. In the first control group, larvae were injected with 10 μL of PBS to monitor the impact of physical trauma. The second control group did not receive any treatment (control of general viability). After injection, the larvae were kept in Petri dishes without nutrition in the dark at 37 °C, and mortality was monitored at 24 h, 48 h, 72 h, and 96 h p.i. Larvae were considered dead if they displayed no movement in response to touch. The statistical significance of differences in the killing of *G. mellonella* larvae by *S. aureus* was assessed by a Gehan-Breslow-Wilcoxon test where a *P* < 0.05 was considered significant.

## Results

### Strain characterization by whole-genome sequencing, pangenome, phylogenetic reconstruction, and GWAS

The selected 95 *S. aureus* BI isolates and the 69 GI isolates were analyzed by whole-genome sequencing, showing that these isolates belonged to various STs. The 15 most commonly observed STs were similar for the BI and GI isolates, showing no significant differences (Supplementary Figure S 1 A). Among the BI isolates, the STs 5, 8, and 30 (9.47% each) were the most abundant STs, whereas the STs 15 and 45 (11.59% each) were most abundant among the GI isolates.

**Fig. 1:**
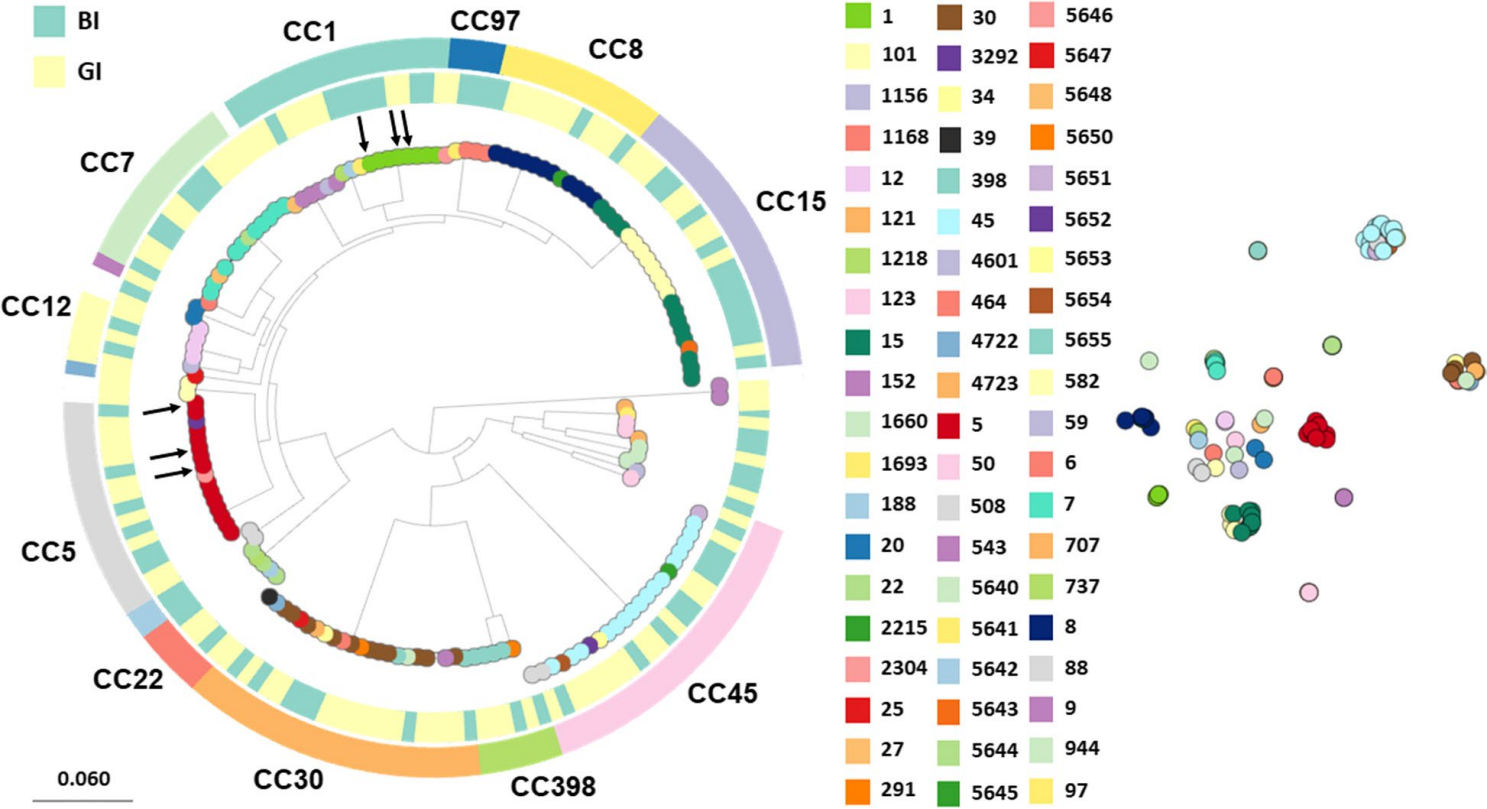
Phylogeny of the 164 BI and GI study isolates. The approximate maximum likelihood phylogeny of the study isolates and the reference strain is shown on the left. Leaves are colored by sequence type as defined in the central legend. The inner ring indicates the isolate source (blue BI, yellow GI), and the outer ring indicates the identified clonal complexes. The arrows indicate the position in the phylogeny of the six isolates that were selected for proteome analyses. The tree and metadata can be found in https://microreact.org/project/5KbM9pyLS5gwpihFXi5ncV-raineri-et-al-2022. Clusters shown on the right are based on the pangenome analysis and were identified with Panini v1.7.1 [1] and Poppunk v2.4.0. using the DBSCAN model

The number of resistance genes per sequenced isolate was low (average 0.87) with *blaZ* being most frequently represented with 5 different variants (Supplementary Figure S 2 B). The average number of identified virulence genes per strain was 62 (Supplementary Figure S 2 C). Twenty-six isolates were *tsst*-1 positive, and only one strain carried the *lukF*-PV and *lukS*-PV genes for the Panton-Valentine leukocidin. No significant differences between carriage versus bacteremia isolates were observed. The pangenome of the entire dataset from 164 *S. aureus* isolates comprised 5535 unique genes. These included 1976 core genes represented in all genomes, consistent with prior core genome estimations [44]. As shown in Fig. 1, the pangenome analysis was in accordance with the core genome clustering, representing several well-defined clonal complexes (CC) of *S. aureus*. These lineages were also identified using the annotation- and alignment-free PopPunk methodology that relies on variable-length-k-mer comparisons ([42]; Supplementary Figure S 2 A). Furthermore, the topology of the tree in Fig. 1 reflects the known population structure of *S. aureus* [15, 46, 87]. The BI isolates were dispersed throughout the tree, which is in accordance with the notion that invasive staphylococcal disease can arise from multiple genetic backgrounds. Interestingly, we detected no significant association between traits and genes using Scoary and Pyseer. This supports the idea that the genetic content of each *S. aureus* isolate (at least the core) is sufficient for the carriage and infection traits [46]. To further assess variations in the entire pangenome content, including intergenic regions, we performed an analysis using IGRs, SNPs, and unitigs. However, based on the results summarized in Supplementary Figure S 1, we conclude that the few suggested associations are insignificant or related to assembly artifacts. No unitigs were found to be significantly associated with carriage or infection.

### Extracellular proteome analyses

Following the genome analysis of the strains, we asked the question whether the extracellular proteomes of representative GI and BI *S. aureus* isolates would show any informative variations that could be related to enteric carriage or infection because the exoproteome is the main reservoir of staphylococcal virulence factors [72]. To this end, we selected isolates belonging to CC1 and CC5, because such isolates were frequently encountered among the characterized GI and BI isolates (Fig. 1; Supplementary Table 1A) and because of the global importance of the respective CC’s. Among the CC1 and CC5 isolates, we selected isolates with the most common *spa* types, which were t127 for CC1 and t002 for CC5. The selected *S. aureus* strains were cultured in RPMI medium until stationary phase (OD_600_ of approximately 1.2), and the proteins in the growth medium were subsequently identified and quantified by MS. RPMI medium was used, because we have previously shown that the global transcript profiles of bacteria grown in human plasma or RPMI are highly similar [48]. Furthermore, extracellular proteins were collected in the stationary phase, because the majority of virulence factors are produced during this growth phase [57]. Interestingly, as shown by LDS-PAGE, the banding patterns of extracellular proteins and their relative intensities were distinct, even for isolates with the same sequence type (ST1 or ST5) (Supplementary Figure S 4 A).

Specifically, the MS analysis of the extracellular proteome identified a total number of 894 proteins, of which 234 proteins were shared between all strains (Supplementary Figure S 3 A). The ST5 isolates shared 264 extracellular proteins amongst each other, whereas the ST1 isolates shared 431 extracellular proteins (Supplementary Figure S 3 B). Furthermore, the BI isolates shared 283 extracellular proteins amongst each other, and the GI isolates 330 (Supplementary Figure S 3 C). The numbers of uniquely identified extracellular proteins also varied for the different strains, irrespective of the sequence type, or site of isolation (Supplementary Figure S 3).

**Fig. 2:**
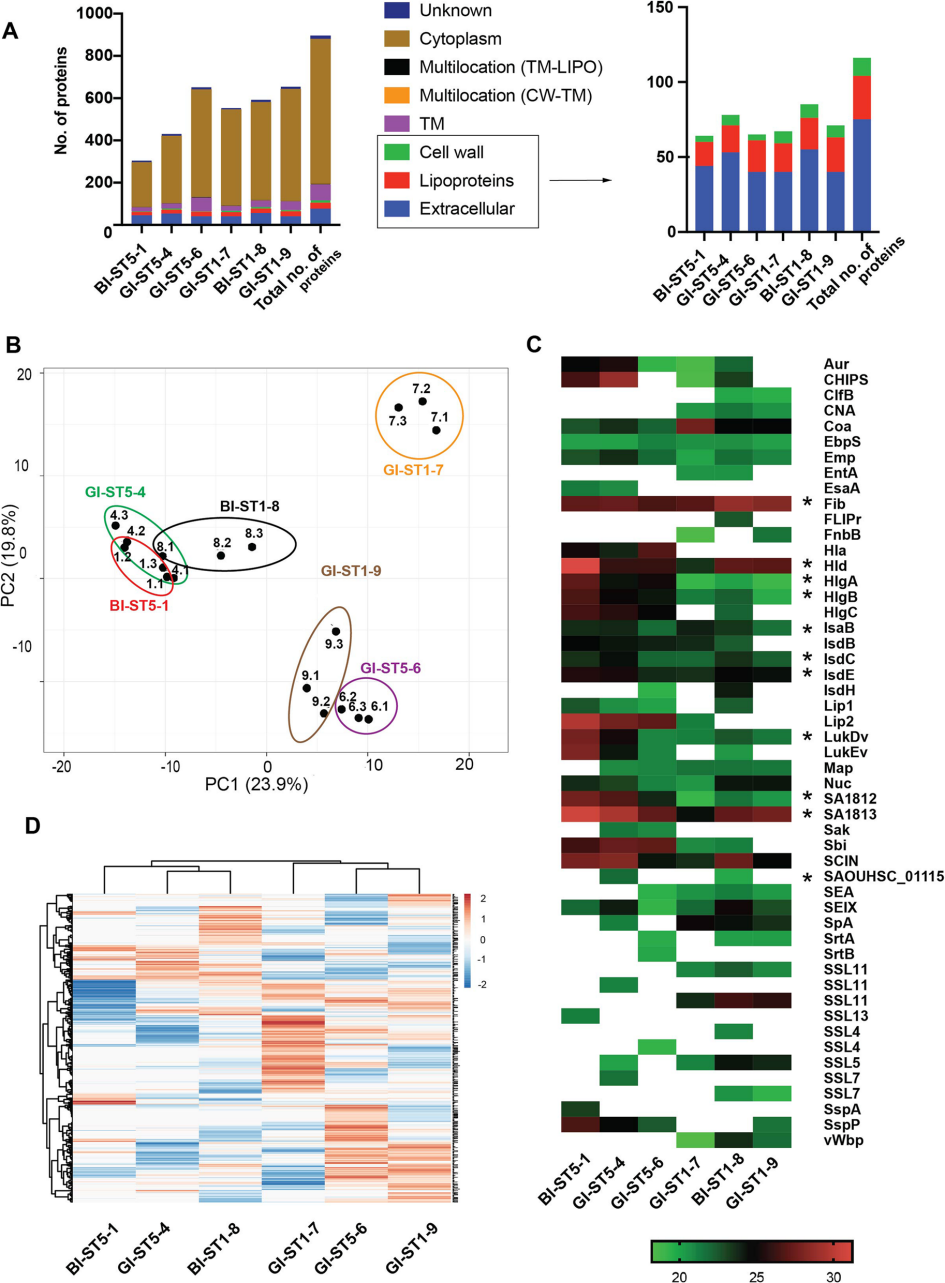
*S. aureus* isolates display high extracellular proteome heterogeneity independently from sequence type or isolation site. **A** Numbers of identified extracellular proteins of the six investigated *S. aureus* isolates and their predicted subcellular locations. For all identified extracellular proteins of the investigated strains, the subcellular locations were predicted bioinformatically with GP4 [23]. **B** Principal component analysis (PCA) based on the LFQ intensities of identified extracellular proteins. The PCA analysis was performed on the identified extracellular proteins of all six *S. aureus* study isolates. Of note, each strain is presented with the 3 replicates separately and the PCA with the averaged values is presented as Supplementary Figure S 4. Note that the small numbers above each data point refer to the respective replicate measurement. **C** Extracellular virulence factors of the six investigated *S. aureus* isolates as identified by proteomics. A total number of 47 virulence factors was identified. Color-coded bars represent the identified proteins and their relative abundance based on the LFQ intensities. *Statistically significant differences in the LFQ intensity of the proteins as assessed ANOVA p < 0.01. Please note that SSL4, SSL7, and SSL11 are listed several times due to significant differences in the respective amino acid sequences. **D** Heat map based on the LFQ intensities of identified extracellular proteins. Of note, the data for each strain are presented as the average of three replicates. The respective data are presented in Supplementary Table S 1

For all identified extracellular proteins, we verified the predicted subcellular localization using the “Gram Positive Protein Prediction Pipeline” GP4 [23]. This revealed that the largest number of identified proteins belong to the so-called class of extracellular cytoplasmic proteins (ECPs), which lack known targeting signals for export from the cytoplasm (Fig. 2A). The number of ECPs was lowest for the BI-ST5-1 isolate and highest for the GI-ST1-9 isolate. The number of extracellular proteins with predicted signal peptides for export via the general secretory (Sec) pathway was around 50 per isolate with a total protein number of 75 signal peptide-bearing extracellular proteins being identified for all investigated isolates. These included 29 predicted lipoproteins and 12 predicted cell wall-associated proteins. Additionally, 4 proteins were predicted to reside at multiple subcellular locations. The high numbers of ECPs identified for the investigated *S. aureus* strains is in accordance with other extracellular proteome studies, which showed that the bacterial “exoproteome” may contain an extensive amount of ECPs [22, 28, 88, 89]. Several identified ECPs belong to the class of “moonlighting proteins” with distinct roles at multiple cellular and extracellular locations, including motility, biofilm formation, host invasion, immunomodulation, and platelet aggregation (Supplementary Fig. 4 C) [22, 28].

A principal component analysis (PCA) based on the LFQ intensities of identified extracellular proteins was performed to elucidate the extracellular proteome relationships among the investigated isolates with different STs and sites of isolation (Fig. 2B). This revealed a high degree of heterogeneity between the isolates, even if they had the same sequence type or were collected from the same site of isolation. Furthermore, the isolates BI-ST5-1, BI-ST1-8, and GI-ST5-4 clustered together, as was the case for the GI-ST5-6 and GI-ST1-9 isolates, whereas the GI-ST1-7 isolate was the most distinct in terms of identified extracellular proteins. To further compare the strains, we generated a heatmap based on the LFQ intensities of the identified extracellular proteins, which further highlights the expression heterogeneity observed for the different proteins, which was independent of the sequence type or isolation site (Fig. 2D).

Our proteome analysis identified 47 proteins that play known roles in the virulence of *S. aureus*. The relative abundance of these proteins per strain is presented in a heatmap (Fig. 2C), and this analysis revealed that the expression of these virulence factors is quite heterogeneous when considering the strains of the same sequence type or from the same isolation site. In fact, only sixteen core virulence factors are expressed by all the investigated strains. The remaining virulence factors show a heterogeneous expression pattern, which is strain-specific. Of note, the presence of the genes for all 47 identified virulence factors was verified using the whole-genome sequences of the six study isolates, which showed that 45 of these genes are present in all the six isolates, while some isolates lack the *cna* (BI-ST5-1, GI-ST5-4, GI-ST5-6) and/or the *chp* genes (GI-ST5-6, GI-ST1-9) (Supplementary Fig. 2 D). The identified virulence factors include proteins with roles in iron acquisition and adhesion to human host cells (IsdB, IsdC IsdE, IsdH), proteins belonging to the “microbial surface components recognizing adhesive matrix molecules” (MSCRAMM) family (ClfB, CNA, EbpS, Emp, Fib, FnbB, Map, and SpA) and sortase enzymes (SrtA and SrtB). Other identified virulence factors are secreted proteins that serve to disrupt host cells and promote spreading, including exoenzymes (Aur, Coa, Lip1, Lip2, Sak, vWpb), proteases (SspA and SspP), and exotoxins (EntA, Hla, Hld, HlgA, HlgB, HlgC, LukDv, LukEv, SA1812, SA1813, SEA, SElX, SSL11, SSL13, SSL4, SSL5, and SSL7). We also identified proteins which are implicated in the evasion of innate or adaptive immune responses of the host (CHIPS, FLIPr, IsaB, Nuc, Sbi, SAOUHSC_01115, SCIN) and a membrane-associated protein of the Type VII secretion system (EsaA). Interestingly, one bloodstream isolate (BI-ST1-8) secreted more known virulence factors compared to the other isolates, while one gut isolate (GI-ST1-9) apparently secreted the lowest number of known virulence factors in comparison to the other isolates. When comparing the secreted virulence factors per sequence type of the isolates, we observed that some virulence factors (collagen-binding protein “CNA” and the von Willebrand binding protein “vWpb”) were present only in the isolates with ST1. On the other hand, the ST5 isolates secreted the staphylococcal α-toxin Hla, which was not identified for the ST1 isolates. None of the extracellular proteins was identified exclusively in gut or bacteremia isolates*.*

**Fig. 3:**
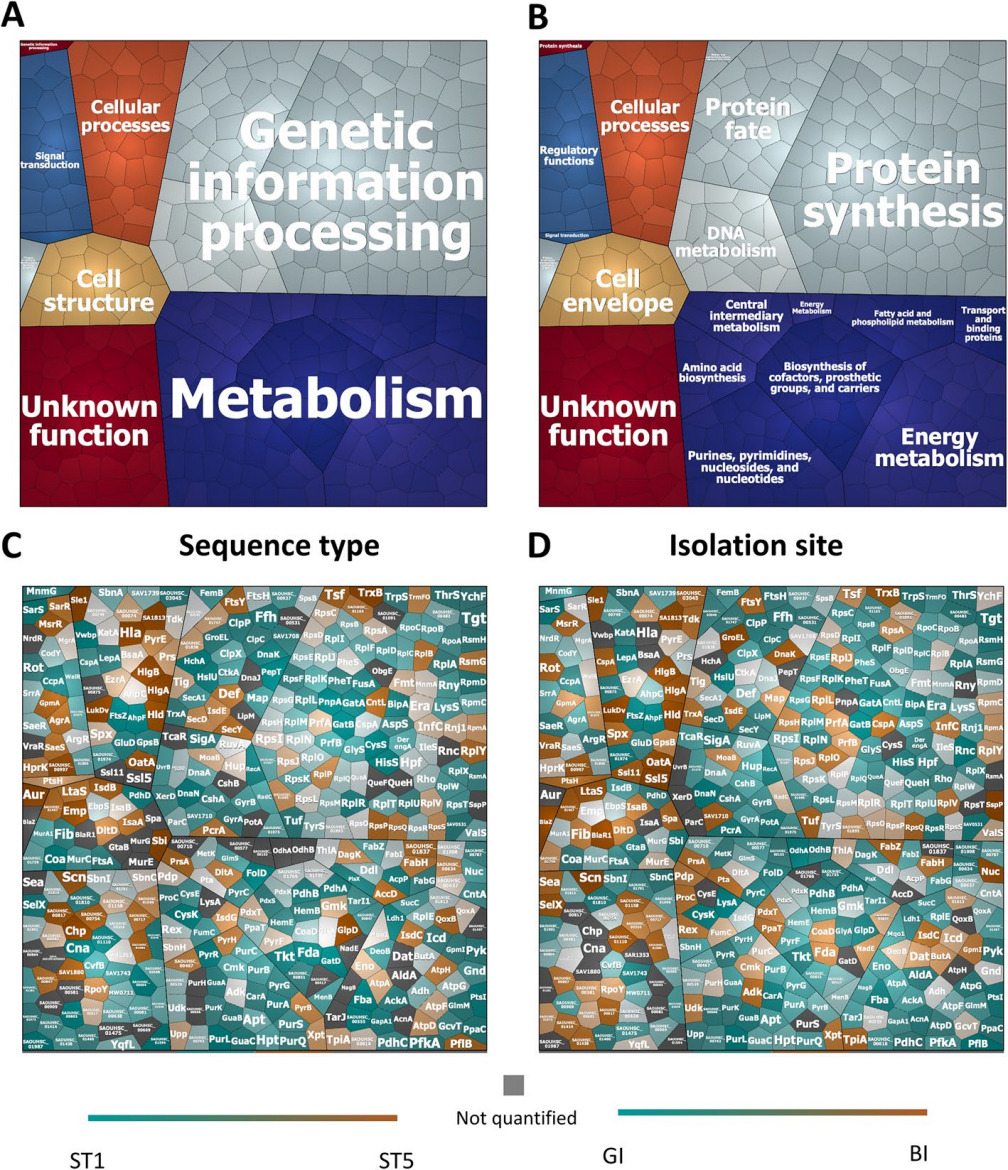
Functional categories and relative abundance of identified extracellular proteins of the investigated *S. aureus* strains. Voronoi treemaps representing the “top-level functions” (TIGRfam level 1) (**A**) and “sub-level functions” (TIGRfam level 2) (**B**). The different functional categories are marked in different colors, and the size of each functional category is proportional to the number of identified proteins with the respective function (**A** and **B**). Each protein is represented by a polygon-shaped tile, and its relative amount was assessed based on ratios of the log2-transformed LFQ intensity values per sequence type (ST5/ST1) (**C**) or per site of isolation (BI/GI) (**D**). Significant differences in the log2-transformed LFQ intensities per sequence type (ST5/ST1) or per isolation site (BI/GI) were assessed by multiple *t* tests and subsequent Holm-Sidak, Bonferroni-Dunn, and Sidak-Bonferroni corrections to adjust the *P* values. Of note, no statistically significant differences were detectable

To determine the overall extracellular proteome functions of the six investigated *S. aureus* isolates, we used the TIGRfam and Aureowiki annotations for the functional classification of the 894 identified proteins [16, 24]. To this end, the proteins were divided into seven top-level functional categories and sixteen sub-level functional categories, as shown in so-called Voronoi treemaps (Fig. 3A, B). In these treemaps, the functional categories are marked in color code and the size of the cells is proportional to the number of identified proteins belonging to the respective category. For the top-level functional categories, the most representative group is composed of proteins with roles in “metabolism” (31.1%), while the remaining groups include proteins involved in “genetic information and processing” (26.6%), “cellular processes” (6.8%), “cell structure” (4.80%), “signal transduction” (4.1%), “phages, prophages, transposable elements, and plasmids” (0.45%) and proteins with unknown function (26.1%) (Fig. 3A). Additionally, the identified extracellular proteins of the six investigated *S. aureus* isolates were compared either by grouping the strains per sequence type (ST5 versus ST1) or per isolation site (GI versus BI). In the respective Voronoi treemaps, each protein is represented by a polygon-shaped tile and its relative abundance is presented based on the log2-transformed LFQ intensities per sequence type (ST5 vs ST1) (Fig. 3C) or isolation site (BI vs GI) (Fig. 3D). Overall, when comparing the identified extracellular proteins per sequence type (ST5 versus ST1) or isolation site (GI versus BI), the proteins belonging to the top-level and the sub-level functional categories do not show significant differences or particular trends. Furthermore, we inspected the unique “ON/OFF” extracellular proteins, which were present (“ON”) in all replicates of one group or absent (“OFF”) from all replicates of the other group. These proteins are Hla, Cna, CysS, GatD, HchA, PepT, PnpA, Vwbp, Ssl11, and SAOUHSC_ (01110, 00094, 00422, 00555, 00603, 01594, 01987, 02447) when comparing the identified proteins per sequence type (i.e., “ON” in ST5/ “OFF” in ST1). The “ON/OFF” proteins compared per isolation site were BlaR1 and BlaZ (“ON” in BI/ “OFF” in GI).

### Cellular proteome analyses

**Fig. 4:**
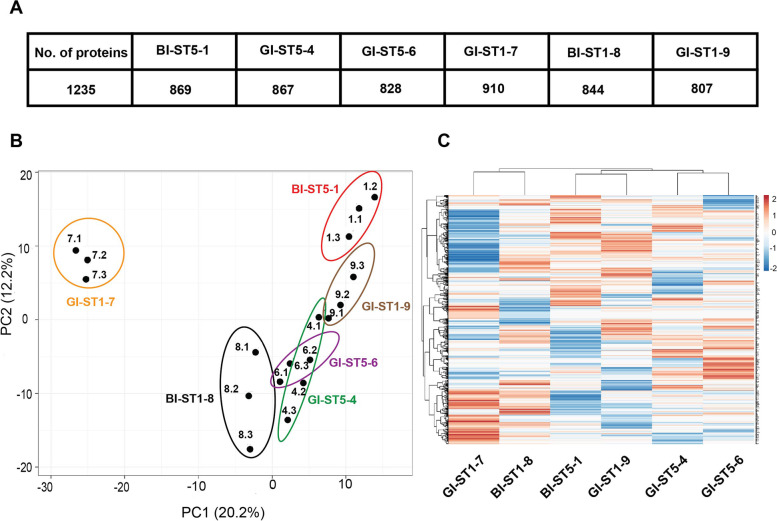
Cellular proteome heterogeneity in the investigated *S. aureus* isolates. **A** Numbers of identified cellular proteins of the six investigated *S. aureus* isolates. **B** Principal component analysis (PCA) based on the LFQ intensities of identified cellular proteins. The PCA analysis was performed on the identified cellular proteins of all six *S. aureus* isolates. The PCA with averaged values is presented in Supplementary Figure S 6. Note that the small numbers above each data point refer to the respective replicate measurement. **C** Heat map based on the LFQ intensities of identified cellular proteins. The data for each strain are based on three independent replicates. The respective data are presented in Supplementary Table S 2

Following the genomic and extracellular proteome characterizations of the six selected *S. aureus* study isolates, we investigated to what extent their cellular proteomes differ. To this end, the strains were cultivated in RPMI medium until the stationary phase (OD_600_ of approximately 1.2). Subsequently, the cells were separated from the growth medium by centrifugation, and the cellular proteins were extracted and identified by MS. This resulted in the identification of 1235 proteins in total, with 610 proteins shared by all isolates (Fig. 4A and Supplementary Figure S 5 A). In particular, the ST5 isolates shared 735 cellular proteins, while the ST1 isolates shared 708 proteins (Supplementary Figure S 5 B). Further, the BI isolates shared 700 proteins, and the gut isolates shared 638 proteins (Supplementary Figure S 5 C). Different numbers of unique cellular proteins were identified for each isolate, irrespective of sequence type or isolation site (Supplementary Figure S 5).

A PCA based on the LFQ intensities of identified cellular proteins was performed to elucidate possible differences between isolates with different sequence types or isolation sites (Fig. 4B). This revealed heterogeneity between isolates with the same sequence type as the three respective ST1 or ST5 isolates did not cluster together. The same phenomenon was observed when the comparison was done per isolation site. In particular, the GI-ST5-4, GI-ST5-6, and BI-ST1-8 isolates cluster next to each other in the PCA, while the BI-ST5-1 and GI-ST1-9 isolates form another cluster and the GI-ST1-7 isolate is distantly positioned in the PCA space. The conclusion that the different isolates are heterogeneous irrespective of sequence type or isolation site is also evident from the heatmap with LFQ intensities of the identified cellular proteins (Fig. 4C).

**Fig. 5:**
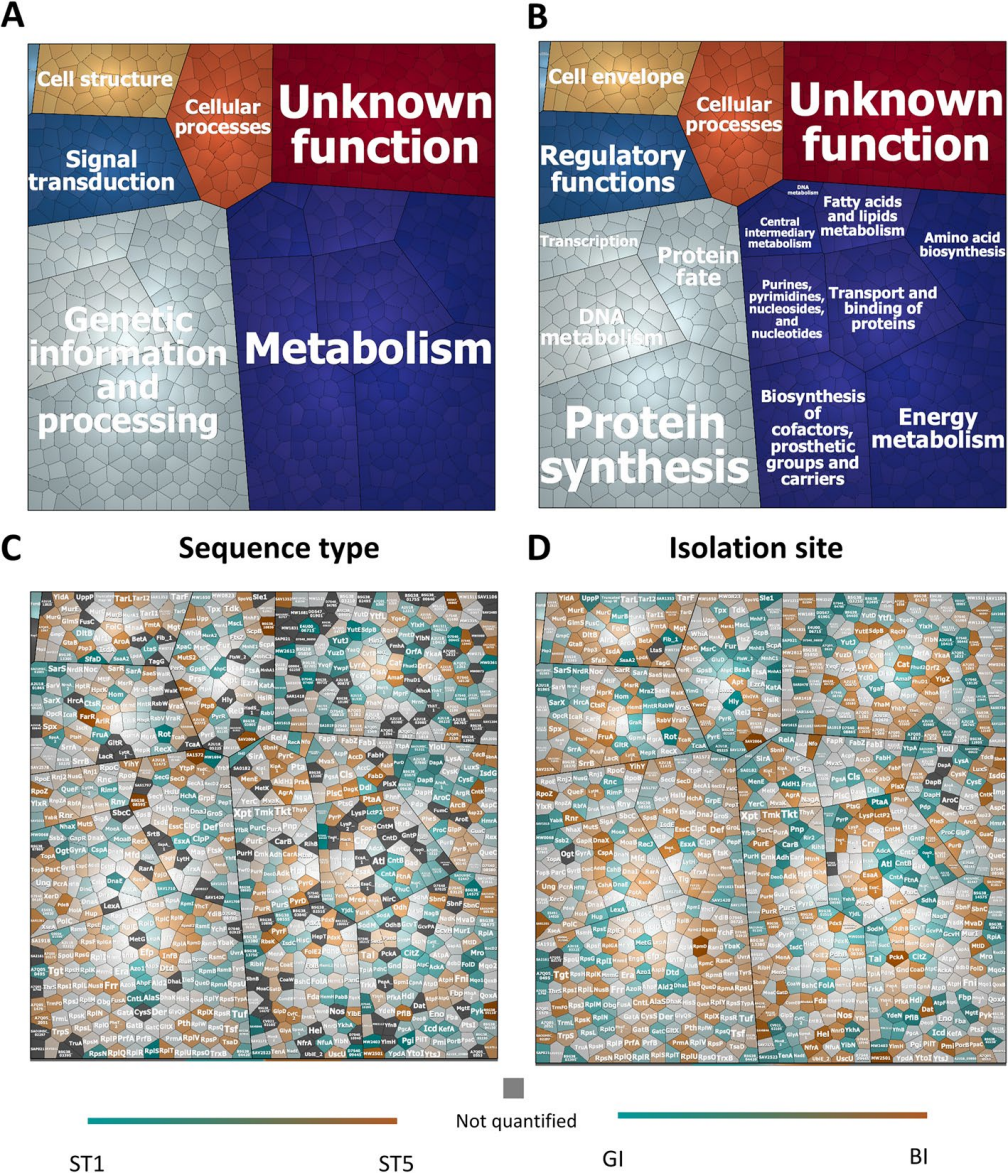
Functional categories and relative abundance of identified cellular proteins of the investigated *S. aureus* strains. Voronoi treemaps representing the “top-level functions” (TIGRfam level 1) (**A**) and “sub-level functions” (TIGRfam level 2) (**B**). The different functional categories are marked in different colors, and the size of each functional category is proportional to the number of identified proteins with the respective functions (**A** and **B**). Each protein is represented by a polygon-shaped tile, and its relative amount was assessed based on the log2-transformed LFQ intensity values per sequence type (ST5/ST1) (**C**) or per isolation site (BI/GI) (**D**) ratios as exported from MaxQuant. Significant differences in the log2-transformed LFQ intensities per sequence type (ST5/ST1) or per isolation site (BI/GI) were assessed by multiple *t* tests and subsequent Holm-Sidak, Bonferroni-Dunn, and Sidak-Bonferroni corrections to adjust the *P* values. Of note, no statistically significant differences were detectable

The overall cellular proteome functions of the six investigated *S. aureus* isolates were evaluated using the TIGRfam and Aureowiki annotations for the 1235 identified proteins [16, 24]. This allowed the distinction of seven top-level and eighteen sub-level functional categories, as shown by Voronoi treemaps where they are marked in color-coded cells that are proportional in size to the number of identified proteins (Fig. 5A, B. The most representative top-level functional group includes proteins with roles in “metabolism” (37.8%, while the following groups include proteins involved in “genetic information and processing” (24.8%; “signal transduction” (6.0%; “cellular processes” (5.9%; “cell structure” (4.9%; “phages, prophages, transposable elements, and plasmids” (0.3%; and proteins with unknown function (20% (Fig. 5A). Overall, when comparing identified cellular proteins per sequence type (ST5 versus ST1) or isolation site (GI versus BI), these proteins belonging to the top-level and the sub-level functional categories do not show significant differences or particular trends (Fig. 5C, D). Additionally, we compared the relative quantities of identified cellular proteins per sequence type (ST5 versus ST1), revealing merely 10 proteins with statistically significant increased abundance in ST5 isolates (Dat, FarR, RecA, SA1975.1, RbsK, D7S40_10290, SAV2523, AroA, AroA_1, and GlyA). More importantly, a comparison per isolation site (BI versus GI) revealed no statistically significant differences in cellular protein abundance between GI and BI isolates. We also inspected the unique “ON/OFF” proteins, which included the SfaD, YutE, RplS, FumC, and Pgi proteins in comparisons per sequence type (“ON” in ST5/ “OFF” in ST1). On the other hand, in comparisons of identified cellular proteins per isolation site (BI versus GI), no unique “ON/OFF” proteins were identified.

*S. aureus* is well known for its great adaptability to different environments by responding to different stimuli. To this end, the bacterium makes use of a range of transcriptional regulators that determine the expression of virulence factors and/or particular metabolic pathways [33, 74]. Here, it should be noted that many staphylococcal genes are actually controlled by multiple regulators. To appreciate the observed differences in protein abundance, we also prepared Voronoi treemaps and heatmaps in which the charted proteins are attributed to the known staphylococcal regulons (Supplementary Figure S 4 and S 6) [55, 56]. In particular, to detect potential metabolic adaptations in the six *S. aureus* isolates in relation to their epidemiology, we categorized the identified proteins according to their involvement in different metabolic pathways, e.g., central carbon metabolism, amino acid metabolism, alternative carbon sources, and respiration as previously described (Palma [51, 63]. The central carbon metabolism, gluconeogenesis, the pentose phosphate pathway, and the tricarboxylic acid (TCA) pathway are essential for *S. aureus* both outside and within host cells [19]; Palma [63]. Additionally, *S. aureus* may have to compete for nutrients when interacting with the human host, which forces *S. aureus* to use amino acids as carbon and nitrogen sources [25], Palma [63], or alternative carbon sources, such as glycerol. Intracellularly, basic cellular functions of *S. aureus*, relating to oxidative phosphorylation, were previously shown to be adjusted based on the availability of oxygen, leading to the employment of alternative metabolic pathways like fermentation [17]. However, as evidenced by the heatmaps of log2-transformed LFQ intensities (Supplementary Figure S 6 B), we did not detect any significant distinctive metabolic adaptations by comparing the cellular proteins per sequence type (ST5 versus ST1) or isolation site (BI versus GI). This implies that, from a metabolic perspective, the behavior of the different GI and BI isolates was comparable upon growth in the RPMI medium. Importantly, this conclusion is fully supported by analyzing the cellular protein abundances per regulon, revealing no statistically significant differences for particular regulons per sequence type or site of isolation (Supplementary Figure S 6 C and D). Likewise, no statistically significant differences for particular regulons were detectable per sequence type or site of isolation upon regulon-based stratification of the identified extracellular proteins (Supplementary Figure S 4 D).

Since no systematic differences could be observed between BI and GI isolates in terms of protein expression in vitro, we asked the question of whether some of these strains would display different virulence profiles upon infection of human cells. For this purpose, we compared their infectious behavior in human gut epithelial cells and blood cells.

### Infection of Caco2 gut epithelial cells

**Fig. 6:**
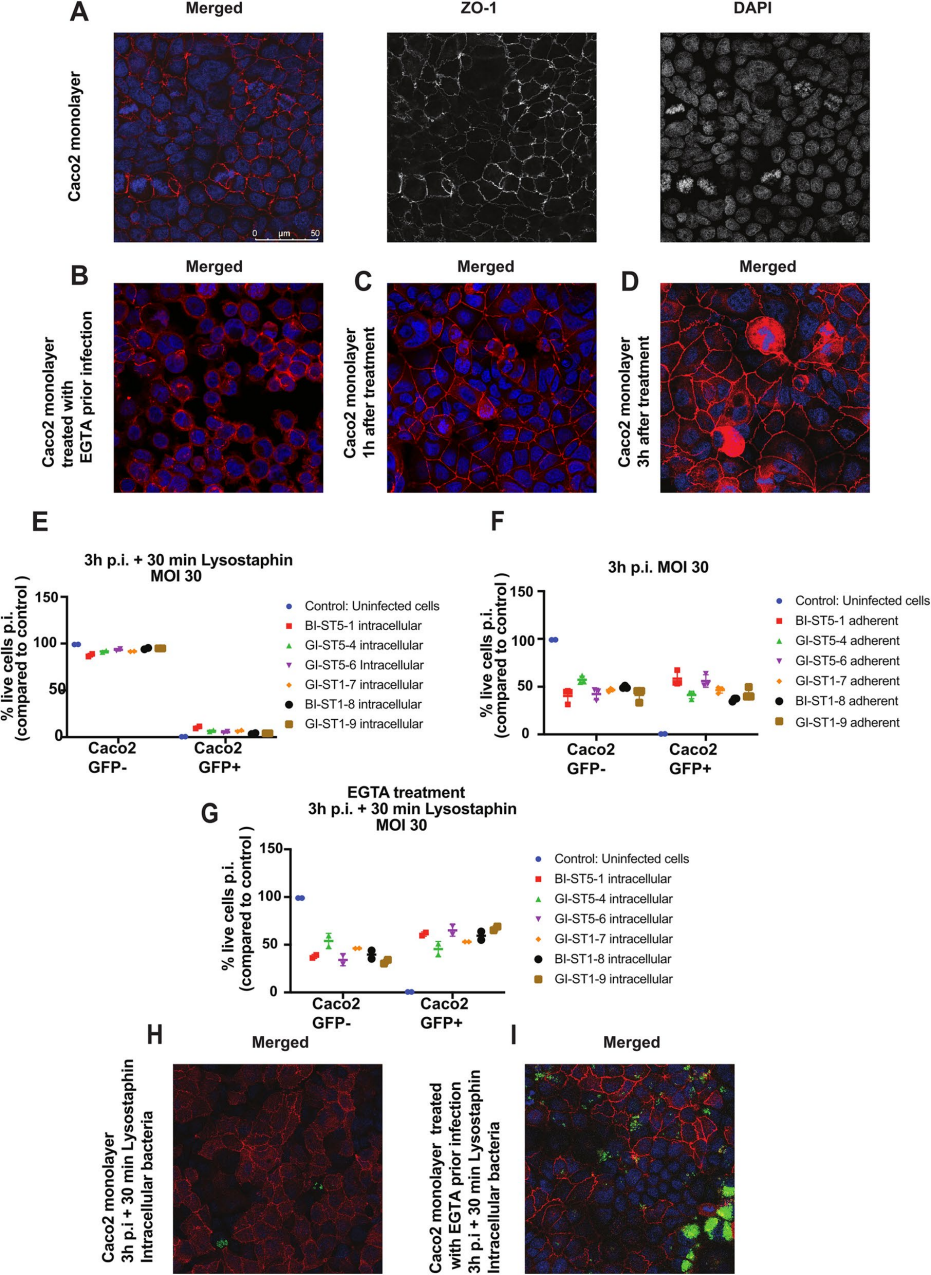
Infection of Caco2 cells by *S. aureus* in the presence or absence of cell–cell junctions. Caco2 cells were seeded in 24-well plates at a density of 200,000 cells per well and cultured for 84 h prior infection. **A** Confocal fluorescence microscopy images of a Caco2 monolayer stained with antibodies specific for ZO-1 (red) and DAPI (blue). Scale bar: 50 µm. **B** Disruption of Caco2 cell–cell junctions by treatment for 45 min with EGTA in calcium-free media. **C** Start of tight junction restoration after EGTA treatment and 1 h incubation in a calcium-free medium and (**D**) complete restoration of tight junctions after 3 h in a calcium-free medium. **E**, **F**, **G** Infection of the Caco2 monolayer or the EGTA-disrupted Caco2 monolayer upon 3 h infection with GFP-expressing *S. aureus*. The percentage of living cells (compared to the uninfected control) that resulted in GFP + or GFP − cells after infection was measured by flow cytometry. GFP + Caco2 indicates the proportion of the cell population with GFP-expressing *S. aureus* and GFP − indicates the population that remained uninfected. **F** Bacterial adherence was measured immediately upon infection in the absence of lysostaphin and, accordingly, GFP + cells represent both adherent and intracellular bacteria. **E**, **G** Upon removal of extracellular bacteria by a 30-min incubation with lysostaphin only intracellular bacteria remain detectable. **H**, **I** Confocal fluorescence microscopy images of Caco2 cells in a closed monolayer (**H**) and upon EGTA treatment (**I**). Cells were stained with an antibody specific for ZO-1 (red) and DAPI (blue). GFP-expressing *S. aureus* are represented in green. Scale bar: 50 µm

To date, little is known about possible interactions between enteric *S. aureus* bacteria and gut epithelial cells, which are likely to be decisive for the transition from the gut-resident to the pathogenic state of this bacterium [67]. Therefore, we established an infection model that is based on monolayers of Caco2 enterocytes, which are simple columnar epithelial cells that line the inner surface of the small and large intestines. Prior to infection, the Caco2 cells were seeded in 24-well plates at a density of 200,000 cells per well and cultured for 84 h prior to infection. This seeding condition was monitored over time by confocal fluorescence microscopy, showing the presence of a monolayer of cells with tight junctions as visualized with antibodies against the ZO-1 protein. Importantly, the Caco2 cells formed monolayers with tight cell–cell junctions at the cellular contact sites (Fig. 6A), mimicking a closed epithelial barrier. Caco2 monolayers were infected with the different *S. aureus* BI and GI isolates for 3 h at a MOI of 30 (Fig. 6C, D), followed by a 30-min incubation with lysostaphin to eliminate non-internalized bacteria. As shown by flow cytometry, only very few bacteria invaded the Caco2 cells, with GFP + Caco2 cells ranging between 4 and 10% (Fig. 6E). Since the Caco2 cell invasion appeared quite homogenous, but low compared to previously investigated endothelial and lung epithelial cell infection models, [62, 63, 68], we investigated whether these low infection levels could be due to a relatively high percentage of bacteria adhering to the cells. To this end, we performed infection experiments where the non-internalized bacteria were not eliminated with lysostaphin, revealing a high percentage of GFP + Caco2 that ranged from ∼35 to 60% (Fig. 6F). The isolates showing the highest Caco2 cell adherence and invasion were the bloodstream isolate BI-ST5-1 and the gut isolate GI-ST5-6. The remaining four isolates showed comparable adhesion to and invasion of Caco2 cells, irrespective of their isolation site or sequence type. This suggested that the tight barrier formed by the Caco2 cells might set a limit to infection. To test this idea, a third Caco2 infection model was established, which mimics a disrupted and subsequently regenerated gut barrier. In this model, the cell–cell junctions of a Caco2 monolayer were temporarily disrupted by a 45-min treatment with EGTA in the absence of calcium (Fig. 6B). Importantly, following the removal of the EGTA, the tight junctions started to be restored after 1 h (Fig. 6C) and after 3 h, the tight junctions were almost completely restored (Fig. 6D). To evaluate the importance of an intact barrier for Caco2 cell infection, we treated the cells with EGTA for 45 min, removed the EGTA by washing, and performed an infection experiment for 3 h. Indeed, following this procedure, we observed a steep increase in the number of bacteria internalized by the Caco2 cells, with GFP + Caco2 cells ranging from ∼35 to 70% (Fig. 6G). The low percentage of GFP + infected Caco2 cells in the monolayer with intact tight junctions (Fig. 6H) compared to the disrupted monolayer after EGTA treatment (F ig. 6I) was also visualized by confocal fluorescence microscopy. Together, these findings demonstrate the importance of a tightly closed monolayer of Caco2 cells in preventing *S. aureus* infection, whereas disrupted cell–cell junctions in the monolayer permit the bacteria to readily enter these gut epithelial cells.

### Leukocyte killing and intracellular survival

**Fig. 7:**
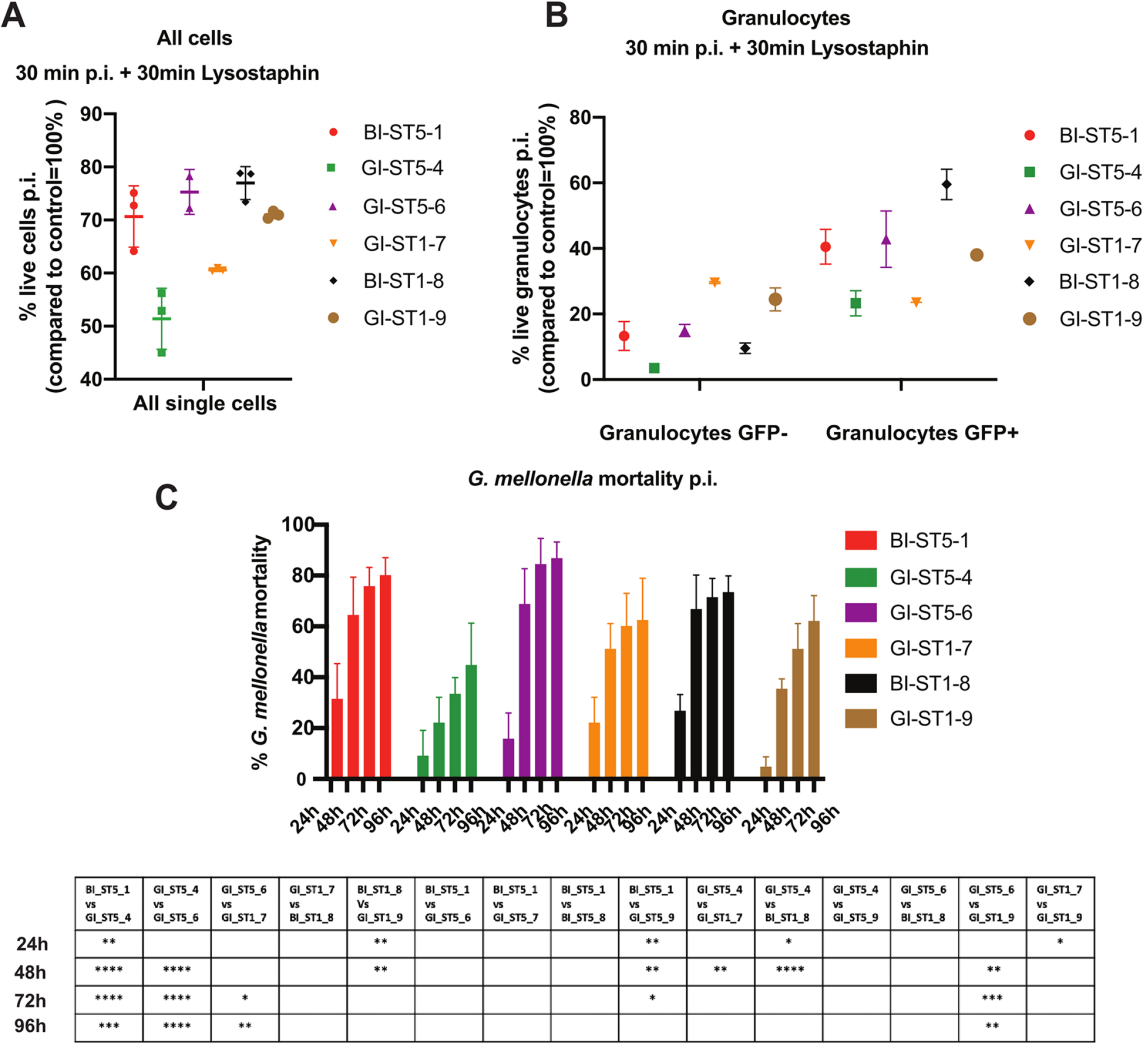
*S. aureus* infection of human leukocytes and larvae of *Galleria mellonella.* **A** Leukocyte infection with the six *S. aureus* study isolates. To investigate the bacterial virulence and intracellular survival after infection of leukocytes, the course of infection was followed by flow cytometry. Per experiment, the percentage of living cells post-infection was measured compared to the uninfected control. **B** Living GFP + and GFP − granulocytes following *S. aureus* infection. GFP + granulocytes indicate the portion of the granulocyte population containing intracellular GFP-expressing *S. aureus*, and GFP − indicates the population that remained uninfected. Cells were infected with *S. aureus* for 30 min and subsequently incubated for 30 min with lysostaphin to remove non-internalized bacteria. **C** Virulence profile of the six *S. aureus* study isolates in *G. mellonella*. To investigate their virulence, three independent *G. mellonella* infection experiments were performed. Per experiment, each strain was used to inoculate 15 *G. mellonella* larvae (45 larvae/strain in total). Each individual larva was inoculated with 10 μl aliquots of a diluted bacterial suspension (1 × 10.^8^ CFU/ml) of the respective *S. aureus* strain. Larval killing was assessed at 24, 48, 72, and 96 h post-inoculation. All values are the mean ± the standard deviation of the three independent infection experiments. Statistical significance of observed differences in virulence between the six *S. aureus* study strains as assessed using a Gehan-Breslow-Wilcoxon test. *P < 0.03; **P < 0.002; ***P < 0.0002; ****P < 0.0001

To gain further insights into the virulence of the six *S. aureus* study isolates once they have entered the bloodstream, we established a model that mimics the interactions between *S. aureus* and blood cells. To this end, we focused our analysis on leukocytes, which are among the first responders once infecting bacteria reach the bloodstream. Moreover, these cells are also present in different human tissues as, for example, the mucosal gut epithelium and the gut lamina propria. The six *S. aureus* isolates were cultured until the stationary phase (OD_600_ of approximately 1.2) and used to infect blood cells collected from healthy volunteers at a MOI of 15. Of note, prior to infection the red blood cells were lysed and removed. Specifically, the bacteria were allowed to interact with the blood cells for 30 min and, thereafter, non-internalized bacteria were eliminated by a 30-min incubation with lysostaphin. Then, the proportion of live blood cells was assessed by flow cytometry (Fig. 7 and Supplementary Figure S 7). As expected, compared to the uninfected control, the percentage of living blood cells decreased upon infection (Fig. 7A). Interestingly, in terms of blood cell killing (around 20 to 30% killing), we observed no difference between the two BI isolates (BI-ST5-1 and BI-ST1-8) and two of the GI isolates (GI-ST5-6 and GI-ST1-9). Moreover, the gut isolates GI-ST5-4 and GI-ST1-7 showed around 40 to 50% killing of blood cells, which means that they are more virulent than the two bacteremia isolates. Of note, these numbers take into account the killing of both monocytes and granulocytes by the infecting bacteria. To assess possible differences in bacterial internalization and intracellular survival in granulocytes, we also measured the percentage of GFP + granulocytes and GFP** − **granulocytes after infection (Fig. 7B). The granulocyte population is mainly composed of neutrophils which, in the human body, continuously migrate through the tissues and are the first responders to bacterial infection. Specifically, the GFP + granulocytes represent those granulocytes that contain intracellular GFP-expressing *S. aureus*, while the GFP-negative granulocytes do not contain bacteria. Infection with one of the BI isolates (BI-ST1-8) led to the highest percentage of GFP + granulocytes, whereas the lowest percentage of GFP + granulocytes was observed for the GI isolates GI-ST5-4 and GI-ST1-7. Altogether, we conclude that the investigated isolates show differing infectious behavior towards human leukocytes that cannot be correlated with their site of isolation or sequence type. Importantly, these observations show that the bacteremia isolates are not necessarily more virulent than the enteric isolates, and they imply that GI isolates may be more pathogenic than isolates that had actually caused an invasive infection in patients.

### Infection of Galleria mellonella larvae

To complement the above infection experiments with human gut epithelial and blood cells with a small animal in vivo model, we infected larvae of the wax moth *G. mellonella* with the six *S. aureus* study isolates*.* Notably, the *G. mellonella* model adheres to the principles of replacement, reduction, and refinement (“3Rs”) and potentially reduces the numbers of vertebrates used for experimental infection studies. In this infection model, the bacteria are challenged primarily by the innate immune system of the larvae*.* The *S. aureus* isolates were cultivated until the stationary phase (OD_600_ of approximately 1.2), and 10 μL of aliquots of each bacterial isolate (1 × 10^8^ CFU/ml) was used to inoculate 45 larvae. The % mortality of *G. mellonella* was subsequently assessed at 24 h, 48 h, 72 h, and 96 h p.i. As shown in Fig. 7C, the different *S. aureus* isolates displayed heterogeneity in larval killing that could not be correlated with their site of isolation or sequence type (Fig. 7C). In fact, infection with the two BI isolates BI-ST5-1 and BI-ST1-8 resulted in a comparable larval killing as was observed for the two enteric isolates GI-ST5-6 and GI-ST1-7, whereas the enteric isolates GI-ST5-4 and GI-ST5-9 were less virulent in larval infections. This finding was conserved over most time points p.i., although the bacteremia isolates tended to be slightly more virulent in the first 24 h p.i. (Fig. 7C). On the other hand, the isolate that caused the highest larval mortality at 96 h p.i. was the gut isolate GI-ST5-6. Therefore, we conclude that, also in the *G. mellonella* infection model, the virulence of our six study isolates cannot be correlated to their site of isolation or sequence type.

## Discussion

*S. aureus* is a frequent member of the human microbiome, as well as a notorious causative agent of bloodstream infections. As such, the diversity occurring within this bacterial species has been studied over many years and at a global scale. Initially, this involved the typing of *S. aureus* isolates based on phages, pulsed-field gel electrophoresis banding patterns, multiple variable tandem repeats in the *spa* gene and different loci as detectable by PCR and/or sequencing, or sequence typing based on variations in particular house-keeping genes at different genomic loci [12, 69]. In recent years, these classical approaches were increasingly complemented with whole-genome sequence-based approaches, which provided deeper insights, not only into the staphylococcal epidemiology and lines of transmission, but also into the evolution of the bacterium following mutation and horizontal gene transfer [26]. These whole-genome comparisons have been instrumental in understanding the emergence and global spread of antibiotic resistance, and the potential roles of mobile genetic elements in virulence. However, they provide relatively little insight into the expression of genes at a genome-wide scale and the actual virulence properties presented by individual isolates. Thus, even though nowadays, there is a vast compendium of well-characterized *S. aureus* lineages of which we know the genetic composition down to the single-base level, and it still remains difficult to predict virulence across different staphylococcal lineages. Clearly, being able to predict virulence based on the genome sequence would be immensely important from the perspective of infection prevention and, indeed, a pioneering study by Laabei et al. managed to predict the virulence of 90 individual MRSA isolates with ST329 based on their genome sequence [38]. Nonetheless, this becomes more difficult for genetically more distant staphylococcal isolates. Consequently, the simple question whether the virulence of, for instance, bacteremia isolates from patients is significantly different from that of colonization or carriage isolates that never caused a noticeable invasive infection in their host was so far still unanswered.

In the present study, we addressed the question whether it would be possible to pinpoint enteric *S. aureus* isolates with a high propensity for causing invasive disease based on particular genomic signatures. To this end, we compared enteric *S. aureus* isolates from healthy human volunteers with blood culture isolates from hospitalized patients with bacteremia. Importantly, our study was based on healthy volunteers and patients from the Northern Netherlands, which represent a fairly genetically homogenous population [75] (https://www.lifelines.nl/). Nonetheless, as shown by whole-genome analyses, the collected *S. aureus* isolates showed substantial diversity, with the isolates belonging to more than 45 different STs. Furthermore, our SNP and unitig analyses revealed no systematic differences between bacteremia and enteric isolates, neither across all sequenced isolates, nor within subgroups of the same sequence type. This also applied to the repertoires of virulence factors and antimicrobial resistance genes, although at this level variations between individual isolates were detectable, which is consistent with results from previous large-scale genome analyses of *S. aureus*. This suggested that there may be no distinctive genomic signatures that separate isolates from the bloodstream and the human gut. This was an unexpected outcome that prompted us to further characterize particular isolates from different sites of isolation and sequence type by proteomics and virulence assays.

In recent years, several genomic- and proteomic-based studies on larger groups of *S. aureus* clinical isolates have shown that isolates classified as highly similar based on genomic signatures may actually present different behavior in terms of virulence and metabolic adaptations [7, 50, 57, 89]. In particular, the first proteomic study on a collection of 25 clinical isolates from one hospital revealed high exoproteome heterogeneity due to genomic plasticity and differences in transcriptional and post-transcriptional regulation [91]. Yet, these isolates were shown to belong to eleven different sequence types, suggesting that the exoproteome variations could relate to the genetic diversity of the investigated *S. aureus* isolates. This view was essentially confirmed by a follow-up study on *S. aureus* isolates with the *spa*-type t437 which, despite the common *spa*-type, represented a genetically heterogeneous population displaying high exoproteome heterogeneity and different pathogenicity [89]. On the contrary, when a homogenous population of USA300 isolates from Denmark was analyzed by comparative genome and proteome analyses, the identified exoproteomes of different isolates were much more similar, though not identical. In fact, based on the differences in the exoproteomes and cellular proteomes of these USA300 isolates, it was possible to distinguish hospital- and community-associated MRSA isolates with distinctive metabolic adaptations [50, 51]. Likewise, distinct proteomic signatures allowed the separation of livestock and human-originated *S. aureus* ST398 isolates [88]. These observations are fully in line with the notion that particular clonal lineages with enhanced pathogenic potential exist, as underscored by the PVL-positive *S. aureus* ST80 or the *S. aureus* ST8 (USA300) ACME + lineages [46, 87]. Together, these previous studies showed that integrated genomic and proteomic analyses can be applied to distinguish groups of genetically closely related clinical *S. aureus* isolates with distinct virulence and epidemiological behavior. In turn, this raised the question whether such an approach could separate related *S. aureus* isolates from the gut or the bloodstream or, vice versa, group isolates from the same site of isolation.

Interestingly, our present study shows that the carriage and invasive behaviors of the investigated *S. aureus* isolates with ST1 or ST5 can neither be separated by genomic nor by proteomic signatures. In fact, our observations show that enteric isolates can be more virulent than bloodstream isolates and vice versa depending on the infection model. Additionally, our results show that the *S. aureus* post-infection events are heterogeneous and dependent on the host cell type. The here-reported differences in infection rates for different host cell types are actually in accordance with previously documented studies that show major differences in *S. aureus* infection of different phagocytic or non-phagocytic cells [30, 73, 76]. Importantly, this is also fully in line with studies showing that the rates of *S. aureus* infections in humans depend also on genetic predispositions, the state of the immune system, and other factors, such as gender, age, ethnicity, and medical history  [54, 67, 70]. One of the decisive factors for invasive staphylococcal disease seems to be barrier disruption. In our present study, we show that gut epithelial cells with an intact barrier were barely infected by *S. aureus* isolates, irrespective of their site of isolation. Only when the cell–cell junctions were disrupted significant invasive behavior was observed, but also in this case with minor differences in infection rates observed for the different study isolates.

Lastly, it should be noted that the *S. aureus* GI isolates characterized in our present study were obtained in a single screening event from stool samples of healthy volunteers. Consequently, we cannot be absolutely certain whether these *S. aureus* isolates represented genuine gut colonizers or transiently carried enteric isolates. For instance, it is conceivable that staphylococci isolated from stool samples originated from nasal or rectal colonization sites. However, irrespective of this possibility, our results show that the investigated *S. aureus* GI isolates have a potential for virulence similar to that of the BI isolates and that a breach of the epithelial cell barrier will provide a facile port of entry for colonizing or carried *S. aureus* bacteria.

## Conclusions

To conclude, here, we show for the first time that *S. aureus* isolates from the human gut microbiome and bloodstream isolates with the same sequence type show no particular signatures at the genomic and proteomic levels that are indicative of a different propensity to cause invasive disease. On the other hand, we show that passage of the gut epithelial layer by enteric isolates or blood isolates is strongly dependent on a disrupted barrier function. These findings are consistent with the view that *S. aureus* is an opportunistic pathogen and that the likelihood of invasive staphylococcal disease is directly depending on the integrity of barriers imposed by epithelial and endothelial layers as well as the underlying immune defenses.

## Supplementary Information

**Additional file 1. Figure S1:** (A) The 15 most common STs found in the *S. aureus* BI and GI isolates collections. (B) Association analysis of IGRs identified by Piggy and with traits based on Scoary and Pyseer. Using the Scoary algorithm, three IGRs were associated with bacteremia, while five were associated with carriage. However, seven of these IGRs were found at the edge of contigs, and one was positioned between two tRNA-encoding genes. We therefore performed the same analysis with Pyseer, using the output from Piggy. This yielded the same results, but in this case five IGRs were tagged with the error ‘bad-chisq’, meaning the χ2 test was invalid and, accordingly, these IGRs were discarded from the analysis. Since there are many tRNAs copies encoded by *S. aureus* genomes, we conclude that this association is most likely an assembly artefact caused by the repetitive nature of tRNA genes. (C and D) Association analysis of SNPs and unitigs with *S. aureus* bacteremia versus enteric isolates. Manhattan plots showing the ‘significant association’ (-log10 P-values) for (C) individual SNPs (significance level 2.80E-06), and (D) individual unitigs (significance level 2.14E-07). Cutoffs for significance are shown by red (C) and blue (D) lines. In accordance with the results from the association analysis of IGRs, no significant associations were identified by applying the SNP and unitig approaches with Pyseer. Six SNPs were associated with the carriage trait (OR=0.23), but these were tagged with the error ‘bad-chisq’ and, therefore, they were discarded from the analysis. The remaining two SNPs were associated with carriage and had an h2=0.372. However, these were located at a recombination site.

**Additional file 2. Figure S2:** (A) Cytoscape network view of the *S. aureus* study isolates using the DBSCAN fit. Nodes (coloured dots) represent samples and edges (lines) represent the pairwise distances classified as within-strain. The nodes are coloured by clonal complexes according to the tree in Figure 1. Resistance and virulence genes of the 218 BI and GI study isolates (B and C). (B) Resistance genes per strain as identified with Abricate using the ResFinder database. Each row represents a strain and in green are depicted the positive strains. The dashed line separates infection isolates (above) from enteric isolates (below). (C) Virulence genes per strain as identified with Abricate using the vfdb database. Each row represents a strain and in blue are depicted the identified genes. The dashed line separates infection isolates (above) from enteric isolates (below). The heatmap on the right depicts the numbers of virulence genes identified for each strain. (D) Virulence factors identified by whole-genome sequencing of the six *S. aureus* BI and GI isolates used for proteome analyses and infection experiments. Please note that SSL4, SSL7, and SSL11 are listed several times due to significant differences in the respective amino acid sequences. Black boxes mark the presence of a particular virulence factor and open boxes mark their absence.

**Additional file 3. Figure S3:** Venn diagrams showing the numbers of common and unique extracellular proteins of the six *S. aureus* BI and GI isolates selected for proteome analyses. The numbers on top of each data point refer to the last number of the strain name. (A) Total number of identified extracellular proteins. Common and uniquely identified extracellular proteins per *S. aureus* sequence type (B) or per isolation site (C).

**Additional file 4. Figure S4:** (A) LDS-PAGE analysis of the exoproteome profiles of the six *S. aureus* strains selected for proteome analyses. (B) Principal component analysis (PCA) based on the LFQ intensities of identified extracellular proteins. The PCA analysis is based on the averaged values of the 3 replicates per strain. (C) ECPs and ‘moonlighting proteins’ of the six *S. aureus *BI and GI study isolates. Color-coded bars represent the identified proteins and their relative abundance based on LFQ intensities. *Statistically significant differences in the LFQ intensities of the proteins assessed by ANOVA (P<0.01). (D) Voronoi treemap representation of *S. aureus* cellular protein levels grouped by regulons. Each protein is represented by a polygon-shaped tile and its relative amount was assessed based on the log2-transformed LFQ intensity values per sequence type (ST5/ST1) or per isolation site (BI/GI) as exported from MaxQuant. Significant differences in the log2-transformed LFQ intensities per sequence type (ST5/ST1) or isolation site (BI/GI) were assessed by multiple t-tests and subsequent Holm-Sidak, Bonferroni-Dunn and Sidak-Bonferroni corrections to adjust the P-values. No significant differences were detected.

**Additional file 5. Figure S5**: Venn diagrams showing the numbers of common and uniquely identified cellular proteins of the six *S. aureus* BI and GI study isolates selected for proteome analyses. (A) Total numbers of identified cellular proteins. Common and uniquely identified cellular proteins per *S. aureus* sequence type (B) or per isolation site (C).

**Additional file 6. Figure S6:** (A) Principal component analysis (PCA) based on the LFQ intensities of identified cellular proteins. The numbers on top of each data point refer to the last number of the strain name. The PCA analysis is based on the averaged values of the three replicates per strain. (B) Assignment of identified proteins according to their roles in metabolic pathways. Color-coded bars represent the identified proteins and their relative amounts as assessed based on the log2-transformed LFQ intensity values per sequence type (ST5/ST1) or per isolation site (BI/GI) as exported from MaxQuant. Significant differences in the log2-transformed LFQ intensities per sequence type (ST5/ST1) or isolation site (BI/GI) were assessed by multiple t-tests and subsequent Holm-Sidak, Bonferroni-Dunn and Sidak-Bonferroni corrections to adjust the P-values. (C and D) Voronoi treemap representation of *S. aureus* cellular protein levels grouped by regulons. Each protein is represented by a polygon-shaped tile and its relative amount was assessed based on the log2-transformed LFQ intensity values per sequence type (ST5/ST1) (C), or per isolation site (BI/GI) (D) as exported from MaxQuant. Significant differences in the log2-transformed LFQ intensities per sequence type (ST5/ST1) or per isolation site (BI/GI) were assessed by multiple t-tests and subsequent Holm-Sidak, Bonferroni-Dunn and Sidak-Bonferroni corrections to adjust the P-values. No significant differences were detected.

**Additional file 7. Figure S7:** Flow cytometry strategies used for infection experiments with cultured Caco2 cells and leukocytes from healthy volunteers. The gating tree was set as follows: FSC-A/SSC-A to represent the distribution of cells in the light scatter based on size and intracellular composition, respectively, to exclude debris; SSC-H/SSC-A to exclude events that could represent more than one cell; SSC-A/FITC-A to select the infected cell population containing GFP-expressing bacteria. (A) uninfected Caco-2 cells. (B) Caco-2 cells infected with GFP-expressing *S. aureus*. (C) Uninfected leukocytes. (D) Leukocytes infected with GFP-expressing *S. aureus*.

**Additional file 8.**

**Additional file 9.**

## Data Availability

All genome sequences have been submitted to GenBank (submission ID SUB11497709) and are available under the accession number BioProject: PRJNA83958. All MS data have been deposited to the ProteomeXchange Consortium via the PRIDE partner repository (Vizcaíno, et al. 2016) with the dataset identifier PXD030971.
