# Supplementary material for: Staphylococcus aureus populations from the gut and the blood are not distinguished by virulence traits—a critical role of host barrier integrity
Source: Microbiome. 2022 Dec 26;10:239. doi: 10.1186/s40168-022-01419-4 (PMC9791742; doi:10.1186/s40168-022-01419-4)

A

BI				GI			
Rank	ST	*spa* types	Frequency (%)	Rank	ST	*spa* types	Frequency (%)
1	5	t002, t045, t179, t306, t311, t539, t653	9.47	1	15	t084, t094, t803, nt	11.59
1	8	t008, t024, t064, t711, nt	9.47	1	45	t026, t065, t116, t282, nt	11.59
1	30	t012, t019, t021, t1827, nt	9.47	2	1	t127, t321, t2207, new	8.70
2	45	t073, t130, t230, t12618, nt	8.42	3	5	t002, t010, t16288	7.25
3	7	t091, t1943, nt	7.37	4	7	t091	5.80
4	582	t065, nt	5.26	5	30	t012, t1456, t16786	4.35
5	15	t1885, t5766, nt	4.21	5	464	t224	4.35
5	398	t6605, t1451, nt	4.21	5	582	t084, nt, new	4.35
6	9	t1546, t3196	3.16	6	8	t008, t16788	2.90
7	1	t127, t1748	2.11	6	12	t156, t160	2.90
7	12	t160, t9259	2.11	6	88	nt	2.90
7	22	t223, nt	2.11	7	9	t1313	1.45
7	101	t056, t1312	2.11	7	20	t16784	1.45
8	6	t304	1.05	7	22	nt	1.45
8	20	t164	1.05	7	34	t6129	1.45

Abbreviations: nt, not typeable by WGS; ST, sequence type.

B

IGR	Gene upstream	Gene downstream	Sensitivity	Specificity	Odds Ratio	Bonferroni P-value (Scoary)
Cluster_6114	tRNA-Ala	tRNA-Ile	0%	67%	0	1.43E-06
Cluster_7925	tRNA-Glu	NA	0%	67%	0	1.43E-06
Cluster_7956	NA	tRNA-Asn	0%	70%	0	1.29E-05
Cluster_6452	tRNA-Ala	NA	97%	38%	19	5.91E-05
Cluster_5792	NA	tRNA-Ala	1%	68%	0	6.81E-05
Cluster_8373	tRNA-Glu	NA	97%	33%	15	2.21E-03
Cluster_5857	5S ribosomal RNA	NA	0%	78%	0	6.85E-03
Cluster_6196	NA	tRNA-Ile	91%	42%	7	1.17E-02

Abbreviations: NA, not applicable.

C

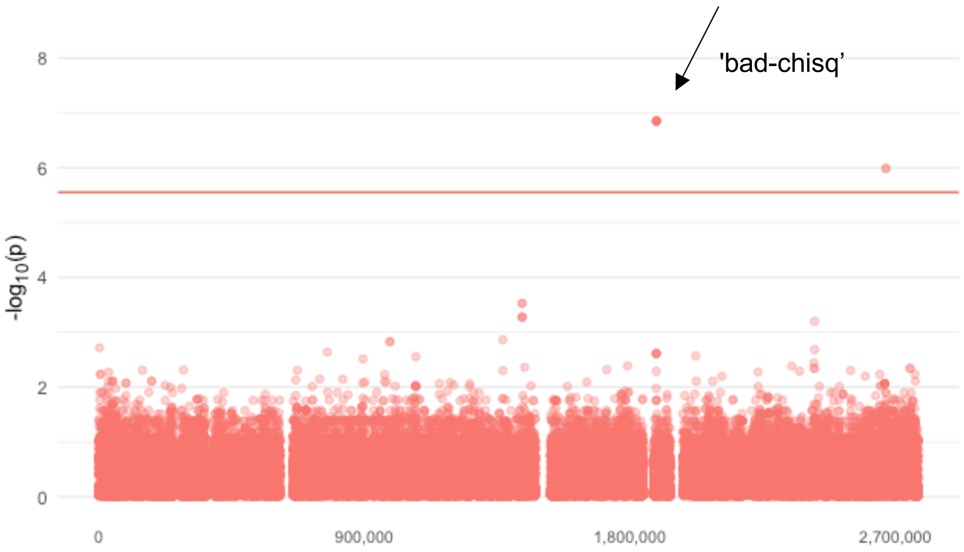

D

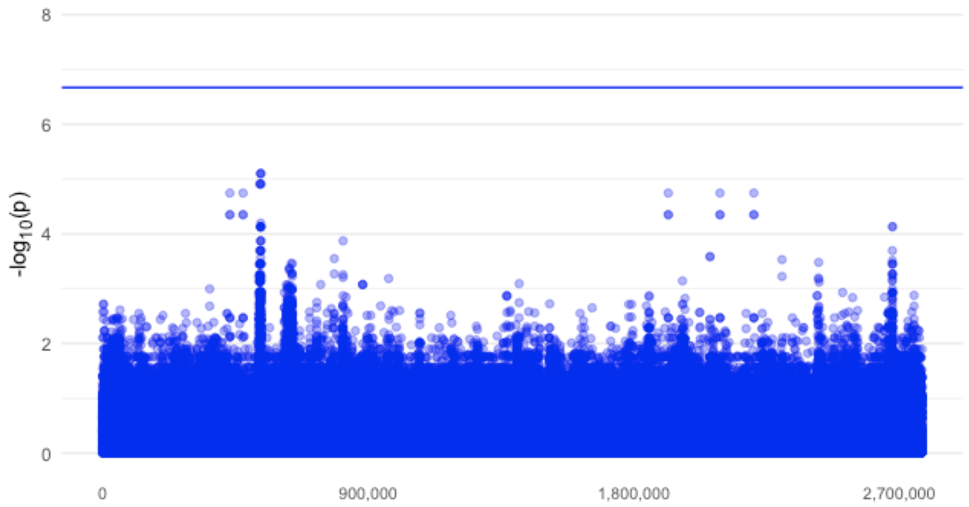

Supplement: Supplementary file 2 — Additional file 1. Figure S1: (A) The 15 most common STs found in the S. aureus BI and GI isolates collections. (B) Association analysis of IGRs identified by Piggy and with traits based on Scoary and Pyseer. Using the Scoary algorithm, three IGRs were associated with bacteremia, while five were associated with carriage. However, seven of these IGRs were found at the edge of contigs, and one was positioned between two tRNA-encoding genes. We therefore performed the same analysis with Pyseer, using the output from Piggy. This yielded the same results, but in this case five IGRs were tagged with the error ‘bad-chisq’, meaning the χ2 test was invalid and, accordingly, these IGRs were discarded from the analysis. Since there are many tRNAs copies encoded by S. aureus genomes, we conclude that this association is most likely an assembly artefact caused by the repetitive nature of tRNA genes. (C and D) Association analysis of SNPs and unitigs with S. aureus bacteremia versus enteric isolates. Manhattan plots showing the ‘significant association’ (-log10 P-values) for (C) individual SNPs (significance level 2.80E-06), and (D) individual unitigs (significance level 2.14E-07). Cutoffs for significance are shown by red (C) and blue (D) lines. In accordance with the results from the association analysis of IGRs, no significant associations were identified by applying the SNP and unitig approaches with Pyseer. Six SNPs were associated with the carriage trait (OR=0.23), but these were tagged with the error ‘bad-chisq’ and, therefore, they were discarded from the analysis. The remaining two SNPs were associated with carriage and had an h2=0.372. However, these were located at a recombination site. [file 40168_2022_1419_MOESM1_ESM.pdf]
