# Supplementary figures and images for: Staphylococcus aureus populations from the gut and the blood are not distinguished by virulence traits—a critical role of host barrier integrity
Source: Microbiome. 2022 Dec 26;10:239. doi: 10.1186/s40168-022-01419-4 (PMC9791742; doi:10.1186/s40168-022-01419-4)

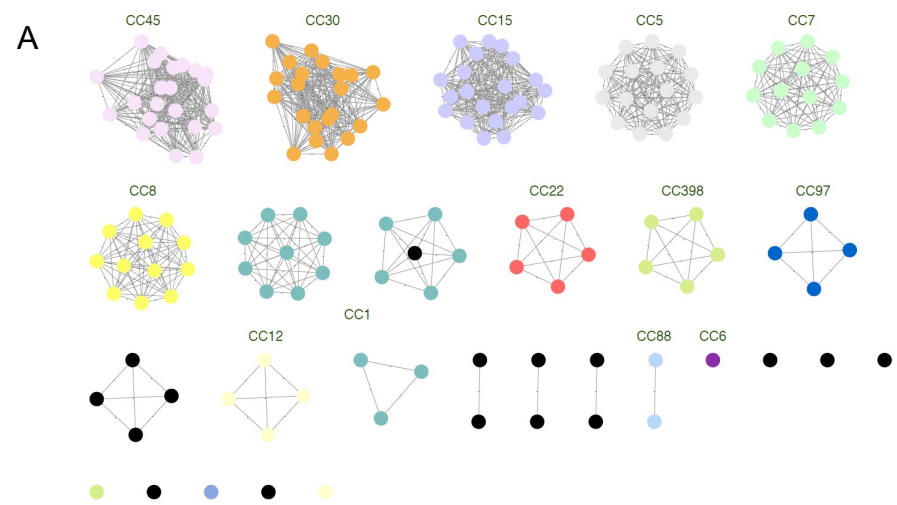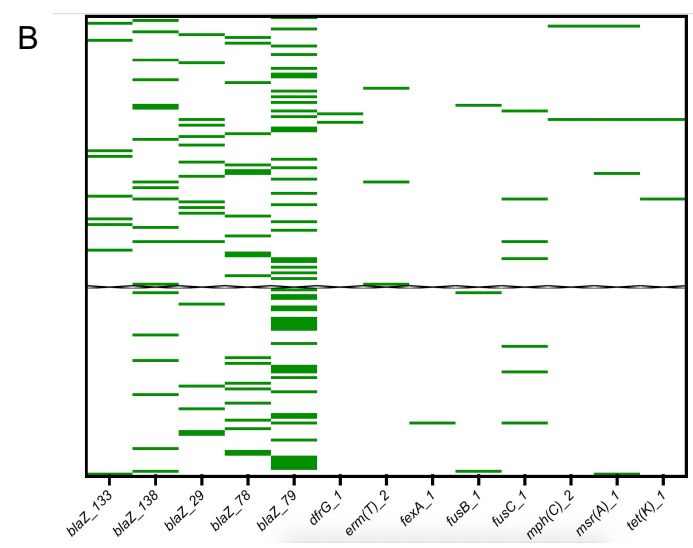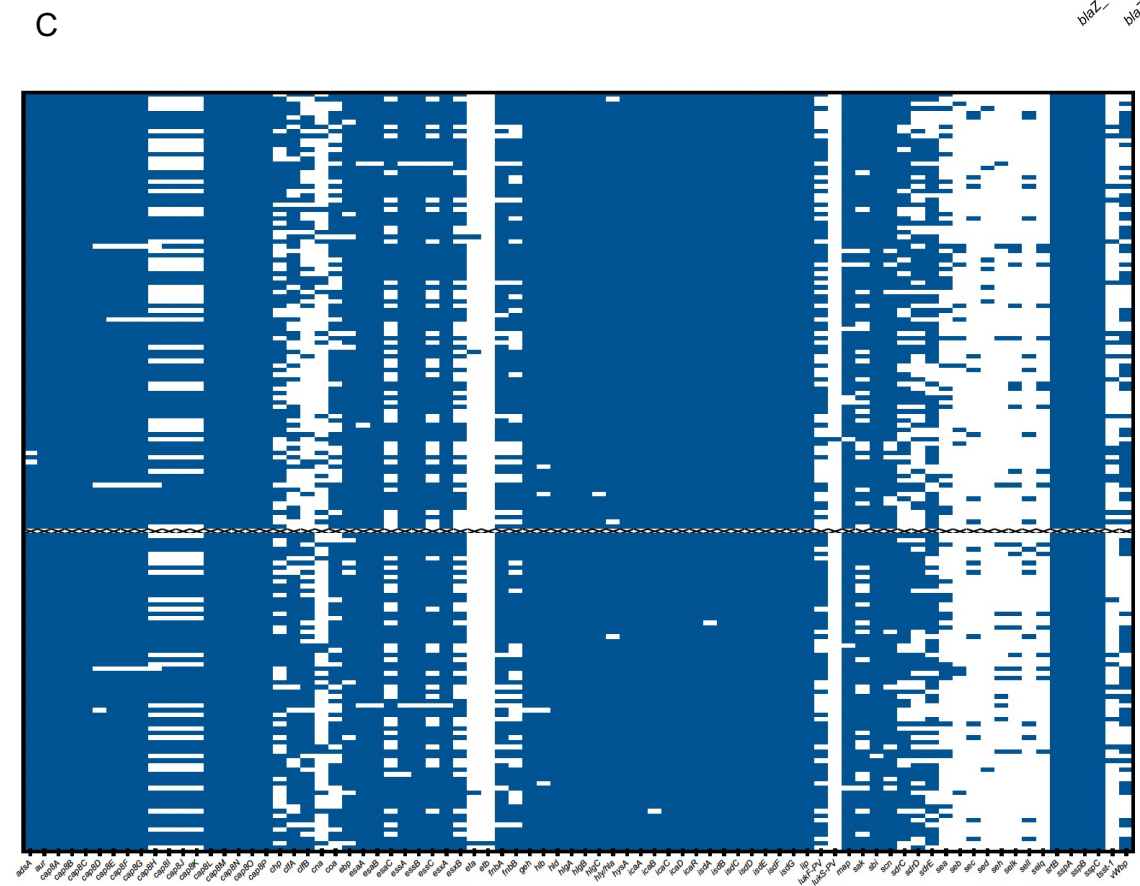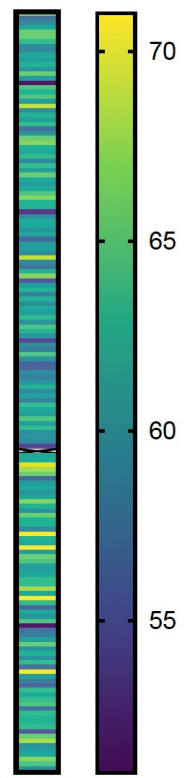

**D**

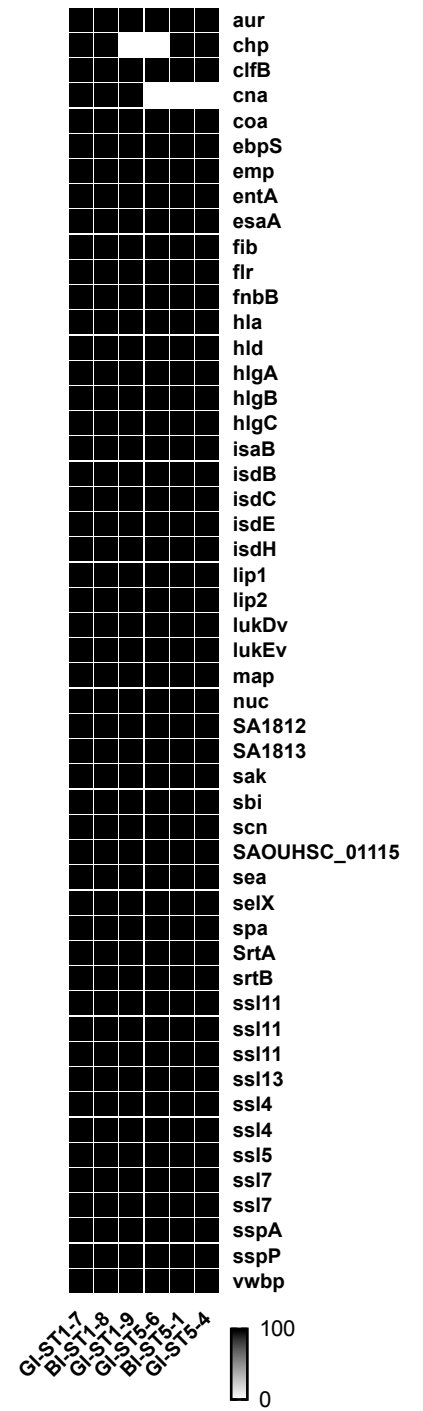

Supplement: Supplementary file 3 — Additional file 2. Figure S2: (A) Cytoscape network view of the S. aureus study isolates using the DBSCAN fit. Nodes (coloured dots) represent samples and edges (lines) represent the pairwise distances classified as within-strain. The nodes are coloured by clonal complexes according to the tree in Figure 1. Resistance and virulence genes of the 218 BI and GI study isolates (B and C). (B) Resistance genes per strain as identified with Abricate using the ResFinder database. Each row represents a strain and in green are depicted the positive strains. The dashed line separates infection isolates (above) from enteric isolates (below). (C) Virulence genes per strain as identified with Abricate using the vfdb database. Each row represents a strain and in blue are depicted the identified genes. The dashed line separates infection isolates (above) from enteric isolates (below). The heatmap on the right depicts the numbers of virulence genes identified for each strain. (D) Virulence factors identified by whole-genome sequencing of the six S. aureus BI and GI isolates used for proteome analyses and infection experiments. Please note that SSL4, SSL7, and SSL11 are listed several times due to significant differences in the respective amino acid sequences. Black boxes mark the presence of a particular virulence factor and open boxes mark their absence. [file 40168_2022_1419_MOESM2_ESM.pdf]

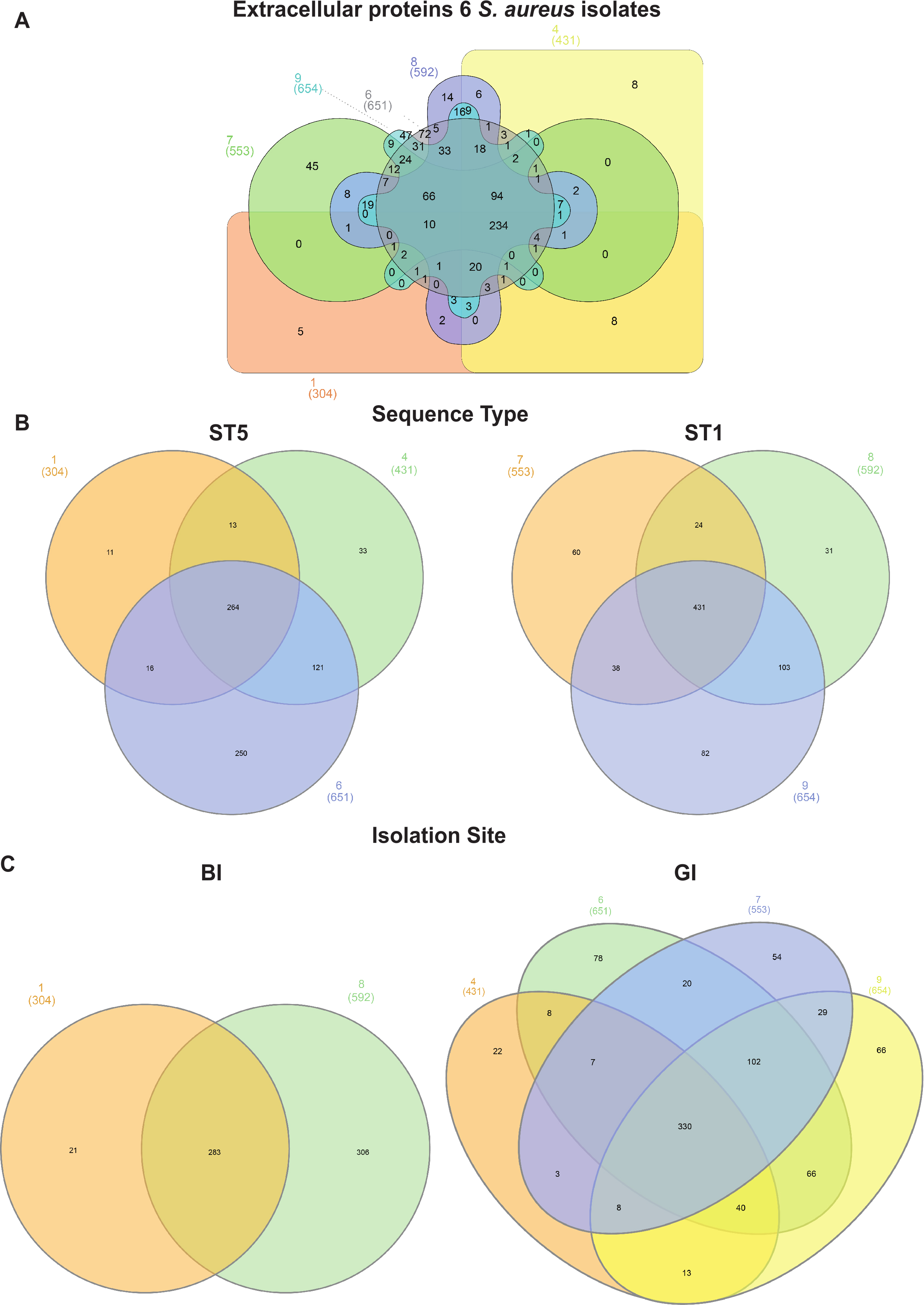

Supplement: Supplementary file 4 — Additional file 3. Figure S3: Venn diagrams showing the numbers of common and unique extracellular proteins of the six S. aureus BI and GI isolates selected for proteome analyses. The numbers on top of each data point refer to the last number of the strain name. (A) Total number of identified extracellular proteins. Common and uniquely identified extracellular proteins per S. aureus sequence type (B) or per isolation site (C). [file 40168_2022_1419_MOESM3_ESM.png]

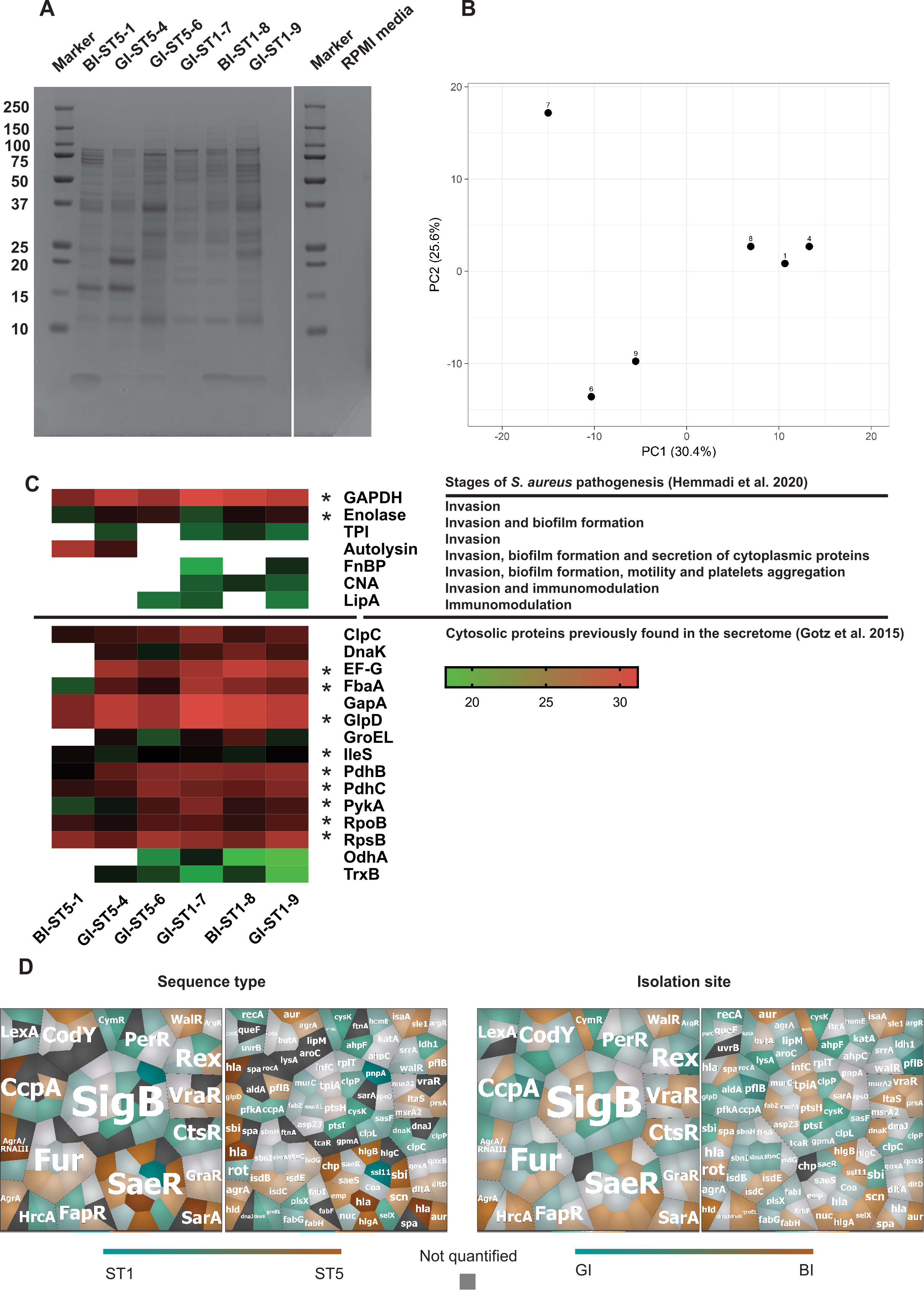

Supplement: Supplementary file 5 — Additional file 4. Figure S4: (A) LDS-PAGE analysis of the exoproteome profiles of the six S. aureus strains selected for proteome analyses. (B) Principal component analysis (PCA) based on the LFQ intensities of identified extracellular proteins. The PCA analysis is based on the averaged values of the 3 replicates per strain. (C) ECPs and ‘moonlighting proteins’ of the six S. aureus BI and GI study isolates. Color-coded bars represent the identified proteins and their relative abundance based on LFQ intensities. *Statistically significant differences in the LFQ intensities of the proteins assessed by ANOVA (P<0.01). (D) Voronoi treemap representation of S. aureus cellular protein levels grouped by regulons. Each protein is represented by a polygon-shaped tile and its relative amount was assessed based on the log2-transformed LFQ intensity values per sequence type (ST5/ST1) or per isolation site (BI/GI) as exported from MaxQuant. Significant differences in the log2-transformed LFQ intensities per sequence type (ST5/ST1) or isolation site (BI/GI) were assessed by multiple t-tests and subsequent Holm-Sidak, Bonferroni-Dunn and Sidak-Bonferroni corrections to adjust the P-values. No significant differences were detected. [file 40168_2022_1419_MOESM4_ESM.png]

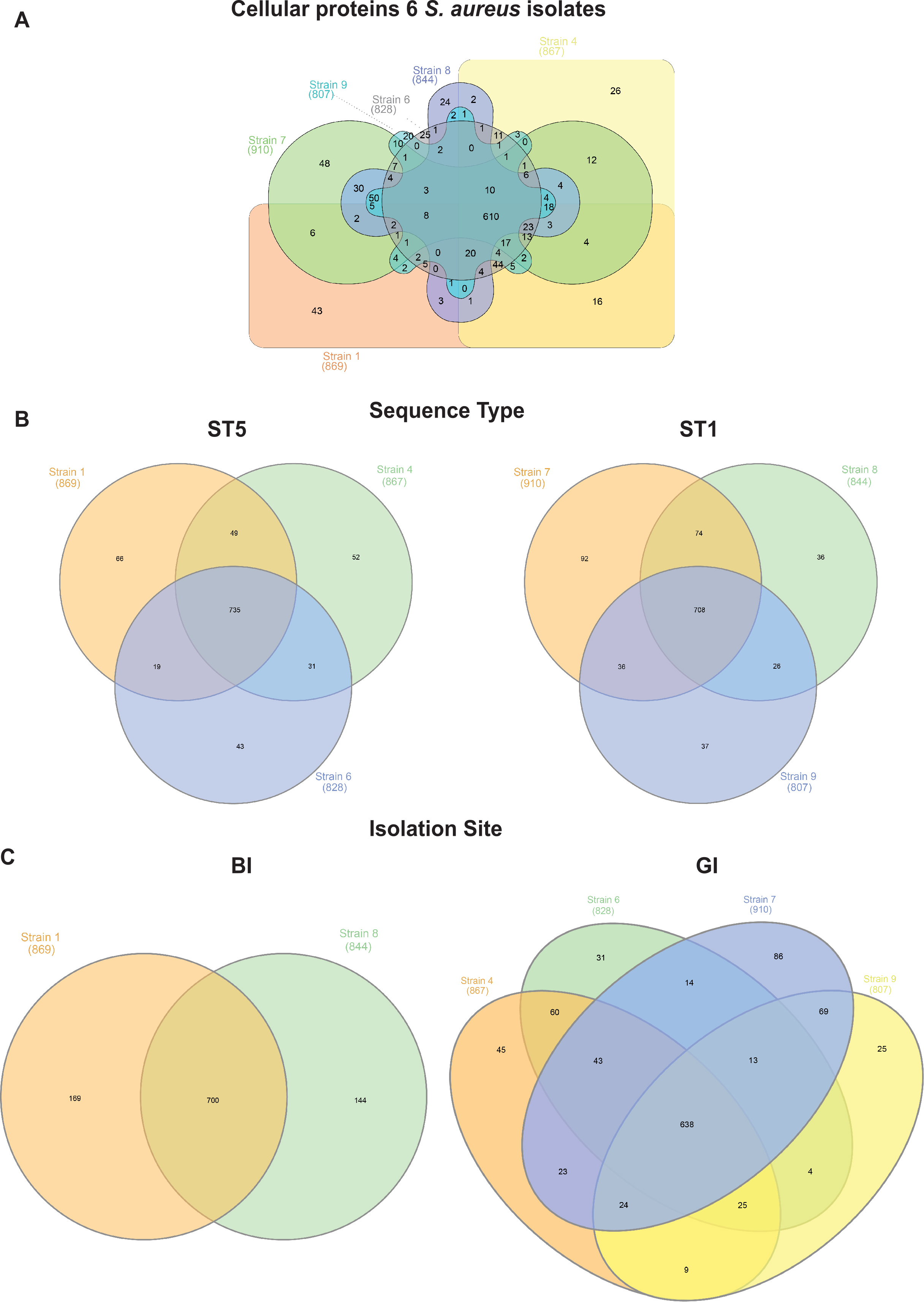

Supplement: Supplementary file 6 — Additional file 5. Figure S5: Venn diagrams showing the numbers of common and uniquely identified cellular proteins of the six S. aureus BI and GI study isolates selected for proteome analyses. (A) Total numbers of identified cellular proteins. Common and uniquely identified cellular proteins per S. aureus sequence type (B) or per isolation site (C). [file 40168_2022_1419_MOESM5_ESM.png]

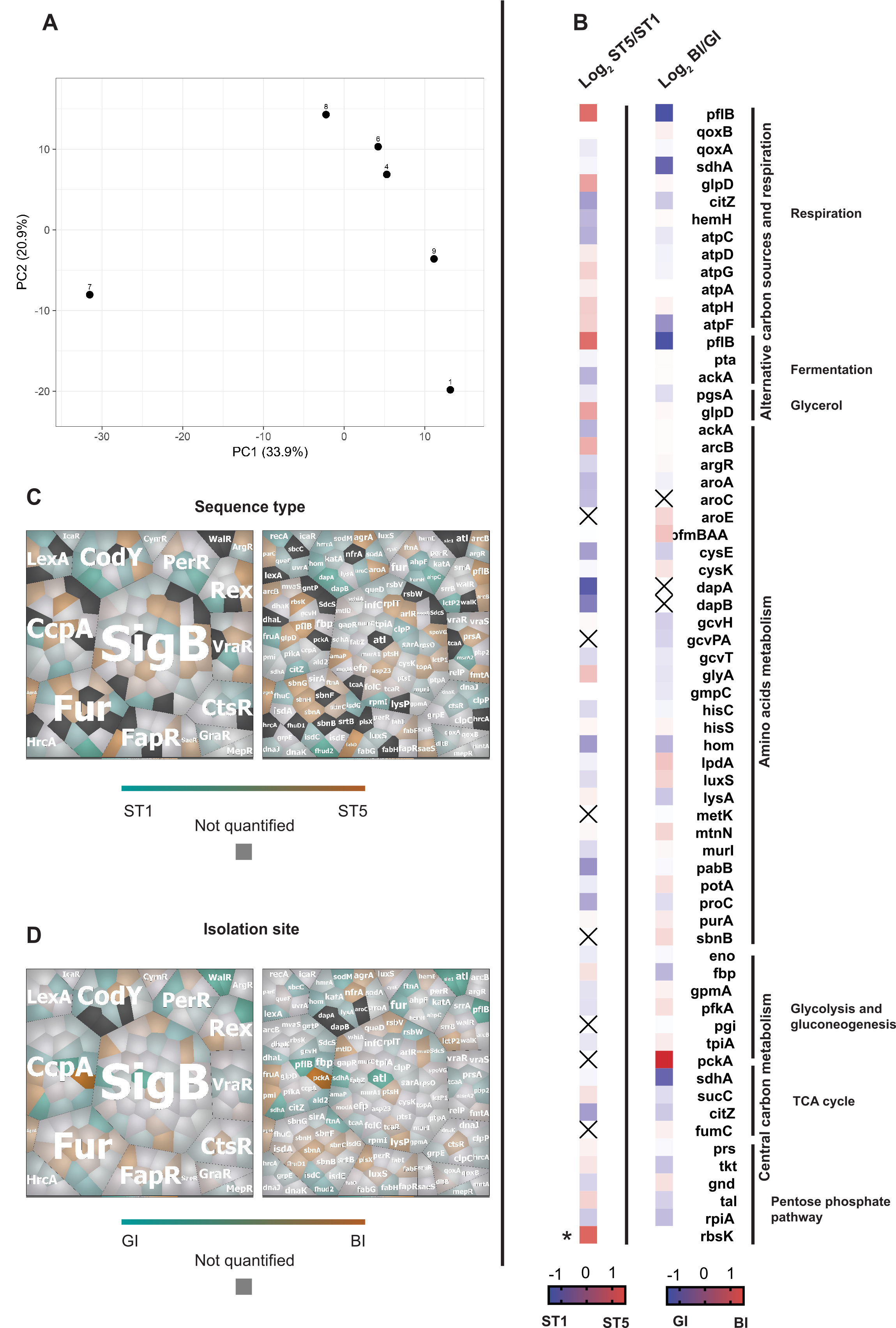

Supplement: Supplementary file 7 — Additional file 6. Figure S6: (A) Principal component analysis (PCA) based on the LFQ intensities of identified cellular proteins. The numbers on top of each data point refer to the last number of the strain name. The PCA analysis is based on the averaged values of the three replicates per strain. (B) Assignment of identified proteins according to their roles in metabolic pathways. Color-coded bars represent the identified proteins and their relative amounts as assessed based on the log2-transformed LFQ intensity values per sequence type (ST5/ST1) or per isolation site (BI/GI) as exported from MaxQuant. Significant differences in the log2-transformed LFQ intensities per sequence type (ST5/ST1) or isolation site (BI/GI) were assessed by multiple t-tests and subsequent Holm-Sidak, Bonferroni-Dunn and Sidak-Bonferroni corrections to adjust the P-values. (C and D) Voronoi treemap representation of S. aureus cellular protein levels grouped by regulons. Each protein is represented by a polygon-shaped tile and its relative amount was assessed based on the log2-transformed LFQ intensity values per sequence type (ST5/ST1) (C), or per isolation site (BI/GI) (D) as exported from MaxQuant. Significant differences in the log2-transformed LFQ intensities per sequence type (ST5/ST1) or per isolation site (BI/GI) were assessed by multiple t-tests and subsequent Holm-Sidak, Bonferroni-Dunn and Sidak-Bonferroni corrections to adjust the P-values. No significant differences were detected. [file 40168_2022_1419_MOESM6_ESM.png]

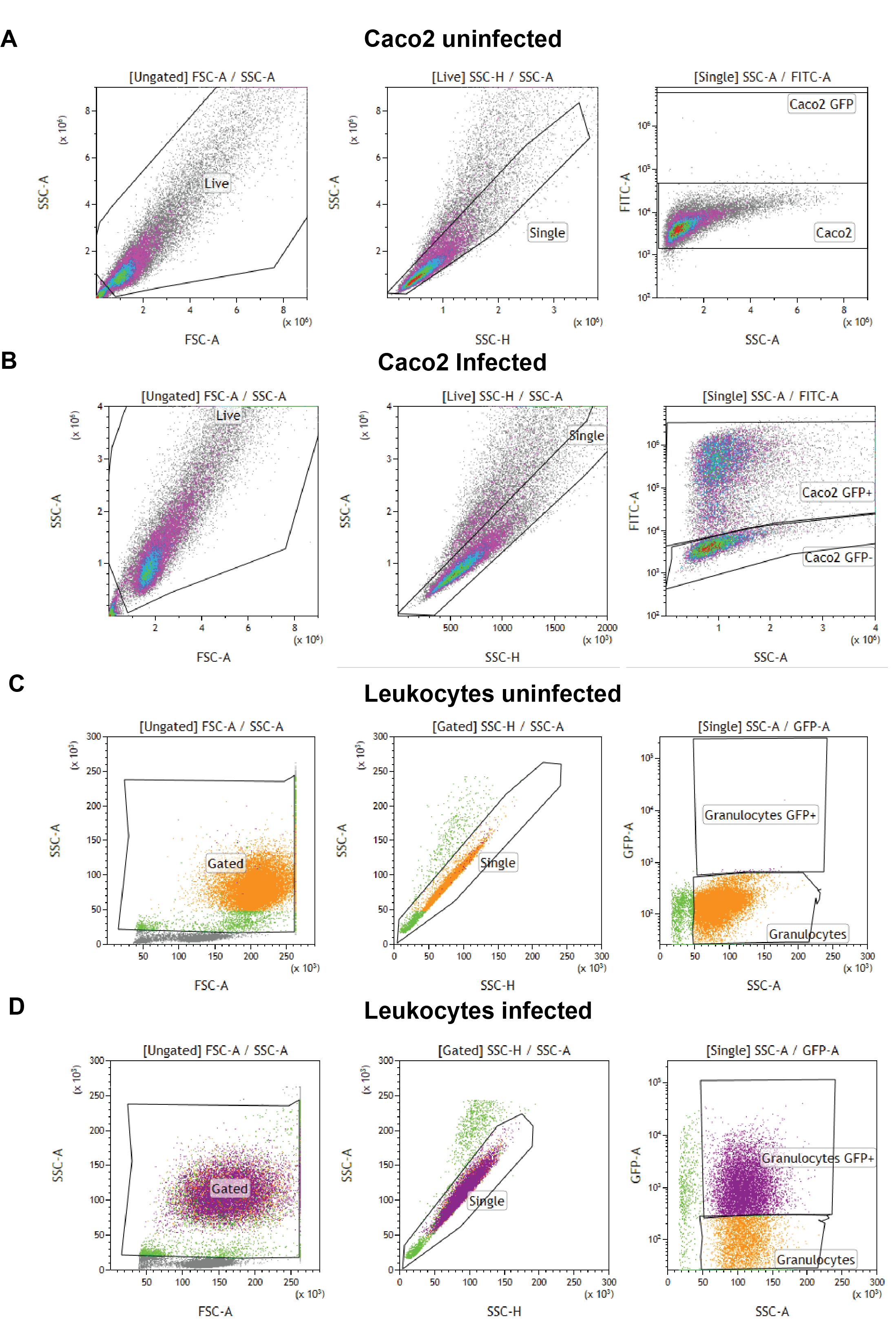

Supplement: Supplementary file 8 — Additional file 7. Figure S7: Flow cytometry strategies used for infection experiments with cultured Caco2 cells and leukocytes from healthy volunteers. The gating tree was set as follows: FSC-A/SSC-A to represent the distribution of cells in the light scatter based on size and intracellular composition, respectively, to exclude debris; SSC-H/SSC-A to exclude events that could represent more than one cell; SSC-A/FITC-A to select the infected cell population containing GFP-expressing bacteria. (A) uninfected Caco-2 cells. (B) Caco-2 cells infected with GFP-expressing S. aureus. (C) Uninfected leukocytes. (D) Leukocytes infected with GFP-expressing S. aureus. [file 40168_2022_1419_MOESM7_ESM.png]
